# Parental and childhood exposures and the risk of retinoblastoma, hepatoblastoma, and germ cell, bone, and soft tissue tumors in children: a systematic review and meta-analysis

**DOI:** 10.3389/fonc.2026.1797553

**Published:** 2026-09-16

**Authors:** Felix M. Onyije, Roya Dolatkhah, Ann Olsson, Liacine Bouaoun, Joachim Schüz

**Affiliations:** Environment and Lifestyle Epidemiology Branch, International Agency for Research on Cancer (IARC/WHO), Lyon, France

**Keywords:** paternal smoking, pesticides, rare cancer, solid childhood cancer, systematic review and meta-analysis

## Abstract

**Introduction:**

Retinoblastoma, hepatoblastoma, germ cell tumors, bone tumors, and soft tissue tumors are rare types of childhood cancer. Identifying modifiable risk factors is essential to inform primary prevention. This review aimed to systematically examine literature and synthesize risk factors for childhood rare cancers.

**Methods:**

PubMed, Web of Science, and Embase databases were searched using lists of keywords and MeSH terms related to parental and childhood exposures. Studies were included if they were case–control or cohort studies of children under the age of 20 years at diagnosis and reported relative risks (RRs) with 95% confidence intervals (95% CIs). Pooled effect sizes (ES) and CIs for risk factors were estimated using random-effects models.

**Results:**

A total of 75 eligible articles from Asia, Europe, Latin America, North America, and Oceania reported cases of rare childhood cancers diagnosed between 1953 and 2021. Birth weight <2,500 g was associated with hepatoblastoma (ES 3.33, 95% CI 1.30–8.54) with considerable heterogeneity across studies. A borderline association was observed for retinoblastoma (ES 1.27, 95% CI 0.99–1.63), while the estimate for germ cell tumors was elevated, albeit with a wide CI (ES 1.20, 95% CI 0.80–1.80). No association was observed for bone tumors or soft tissue tumors. Small for gestation age was associated with hepatoblastoma (ES 1.76, 95% CI 1.28–2.43). Birth weight >4,000 g was not associated with any of the studied cancer types. Assisted reproductive technology (ES 2.82, 95% CI 1.82–4.39), Caesarean section (ES 1.47, 95% CI 1.20–1.79), and parental age >35 years (mothers ES 1.97, 95% CI 1.45–2.68; fathers ES 1.33, 95% CI 0.99–1.79) were observed to be associated with hepatoblastoma. Paternal smoking during preconception/pregnancy was associated with an increased risk of hepatoblastoma (ES = 1.58, 95% CI 1.18–2.12). Paternal occupational exposure to pesticides during preconception/pregnancy was associated with an increased risk of retinoblastoma (ES = 1.55, 95% CI 1.09–2.21). For some other exposures, we found several raised ES but with very wide CIs.

**Discussion:**

We identified only a few modifiable risk factors for certain childhood cancers, notably paternal smoking for hepatoblastoma and paternal occupational exposure to pesticides for retinoblastoma. Larger studies for rare childhood cancers are needed, with international collaboration to increase sample sizes.

## Introduction

Globally, retinoblastoma, hepatoblastoma, germ cell tumors, bone tumors, and soft tissue tumors are considered rare childhood cancers, each with an incidence rate below one per 100,000 children per year (1–6).

Retinoblastoma is an aggressive cancer of the retina and occurs more often unilaterally than bilaterally. It occurs slightly more frequently in male children than in female ones, with an estimated male-to-female ratio of 1.3:1.00. Retinoblastoma typically peaks at 1 to 2 years of age for sporadic (usually unilateral) cases, whereas bilateral retinoblastoma, often heritable, peaks earlier at around 9–12 months (7–9). The 5-year survival rate is approximately 95% in high-income countries (HICs), largely due to early detection and access to chemotherapy (10, 11). In contrast, survival in low- and middle-income countries (LMICs) ranges from 30% to 60%, primarily because of delayed presentation and limited access to treatment (12). Clinically, leukocoria is the most common presenting sign, occurring in approximately 56% of cases, followed by strabismus in approximately 20% (13).

Hepatoblastoma is the most common malignant tumor in the liver accounting for 90% of pediatric hepatic tumors. Countries such as the United States, Japan, and those in Western Europe demonstrate higher reported incidence rates, partly due to more complete surveillance and comprehensive registries (14, 15). It occurs predominantly in children under 5 years of age and shows a male predilection, with an estimated male-to-female ratio ranging from 1.2:1 to 3.6:1 (16, 17). Due to significant medical and surgical advancements in recent years, the overall survival of low-risk hepatoblastoma has improved markedly from approximately 30% to 90%. In contrast, high-risk patients, particularly those with metastatic disease, older age at diagnosis, or bilobar liver involvement, experience much poorer outcomes, often with survival rates below 50%. These outcomes are even lower in LMICs (18, 19).

Unlike the other tumor types, germ cell tumors are a heterogenous group occurring at various anatomical sites. Malignant germ cell tumors are rare, accounting for approximately 3%–5% of childhood neoplasms, with an estimated male-to-female ratio of 0.8:1 (20, 21). Although it is predominantly localized in the gonads, they can also occur in extragonadal sites, particularly in children <6 years (22). Childhood ovarian germ cell tumors show a peak incidence in early adolescence, whereas childhood testicular germ cell tumors exhibit two distinct age peaks: children younger than 3 years, who may present with mature teratomas or malignant germ cell tumors, and adolescents, who may develop seminomas or mixed malignant tumors (23). An increasing incidence of malignant testicular germ cell tumors has been reported in many countries, particularly in Northern and Western Europe. In contrast, incidence trends for ovarian germ cell tumors vary, with studies reporting both increases and decreases over time (21, 24–28). In HICs, testicular germ cell tumors have an excellent prognosis, with an overall 5-year relative survival of approximately 95%. For localized testicular cancer, the 5-year survival rate exceeds 99% (29). Data are sparse for LMICs, with survival rates generally lower.

Malignant bone tumors account for approximately 3% of childhood and adolescent cancers. Osteosarcoma and the Ewing sarcoma family of tumors are the most common malignant bone tumors, together representing the majority of cases (30). Osteosarcoma and Ewing sarcoma in children have a peak incidence in the second decade of life (31–34). The overall male-to-female ratio is approximately 1.3:1. The 5-year overall survival for childhood bone tumors is around 60% in HICs (35–37) but is substantially lower in LMICs, ranging from 20% to 40% (37, 38).

Similarly, soft tissue tumors also peak in the second decade of life. Soft tissue sarcomas account for approximately 7% of all childhood cancers and are broadly classified into two main types: rhabdomyosarcoma and non-rhabdomyosarcoma soft tissue sarcomas.

Rhabdomyosarcoma is the most common soft tissue sarcoma in children and is a high-grade malignancy composed of cells resembling skeletal myoblasts (39). Notably, soft tissue tumors show a higher prevalence in male patients, with an approximate male-to-female ratio of 1.5:1 (40). The 5-year overall survival for malignant soft tissue tumors is around 65% in HICs, compared with approximately 30% in LMICs (41, 42).

The etiology of these rare childhood cancers remains largely unknown. Risk factors investigated in relation to childhood cancers included assisted reproductive technology (ART), Caesarean section (C-section), gestational age, small for gestational age (SGA), large for gestational age (LGA), birth weight, parental age, gestational diabetes, paternal smoking, pesticide use, and parental occupational exposures, all of which have been hypothesized to increase the risk for childhood cancers (43–49). The aim of this systematic review and meta-analysis is to synthesize and elucidate evidence from different epidemiological studies on the risk factors associated with retinoblastoma, hepatoblastoma, germ cell tumors, bone tumors, and soft tissue tumors, which may inform primary prevention. Although the primary focus of this review was on modifiable risk factors, we additionally included other non-modifiable risk factors to provide a more comprehensive overview of factors associated with these rare childhood cancers.

## Materials and methods

### Search strategies and selection criteria

We used the Preferred Reporting Items for Systematic Reviews and Meta-Analyses (PRISMA) 2020 checklist to conduct the systematic review and meta-analysis (50) (**Supplementary Material**, pages 3–5). The search strategy used for article identification and the methods for data extraction and analysis have been previously reported (51, 52). The databases used in this study were PubMed, Web of Science, and Embase, with no restrictions on publication date and language.

### Inclusion and exclusion criteria

We extracted information from included articles published between 1988 and 2024. The studies included were case–control or cohort studies of cancer (retinoblastoma, hepatoblastoma, germ cell tumors, bone tumors, and soft tissue tumors) diagnosed in children and adolescents under the age of 20 years, from which we extracted exposure time windows and estimates of relative risks (RRs), such as odds ratio (OR), hazard ratio, standardized mortality ratio, mortality rate ratio, standard incidence ratio, or incidence rate ratio with 95% confidence intervals (CIs). We checked publications from the same region for overlaps of their study populations. The inclusion and exclusion criteria were defined *a priori* (**Supplementary Material**, page 2) (51, 52).

### Study selection and data extraction

Identified peer reviewed articles were retrieved and screened for duplicates in EndNote version X9.3.3. The authors, FMO and RD, assessed the titles, abstracts, and full text of the articles independently to determine their eligibility (53). Disagreements arising from the independent selection process were resolved by seeking the opinion of the third and last authors, AO and JS. Additional articles were sourced from the lists of references of the selected articles. We structured our search strategy in line with population, exposure, comparator, and outcome (PECO) components and included a list of keywords and MeSH terms (**Supplementary Material**, pages 6–11). The search was initially conducted in June 2022 and subsequently updated until January 2025 (we reran the original search strategy using the same databases and search terms but looking from the end of the previous search to January 2025).

Risk factors extracted included birth and parental characteristics, occupational exposures to pesticides, wood dust, paint, gasoline fumes and livestock/animals, and lifestyle exposures including maternal diet, vitamin intake, parental smoking, and alcohol consumption. Others were maternal medical radiation exposure, residential proximity to major roads, and childhood exposure to traffic density. Exposure time period such as preconceptional, prenatal, and postnatal were also extracted. Other information extracted include author names, year of publication, study location, period and age range of diagnosis, exposure assessment methods, outcome ascertainment, number of childhood cancer cases and controls or, if not available, the study population, follow-up duration, and risk estimates with their respective 95% CIs. Information regarding study design (case–control and cohort or nested case–control) was also extracted. Registry-based case–control studies were considered as cohort design in the present analysis, as usually nested within a nationwide registry landscape and therefore similar, in design, to those case–control studies nested within cohorts (51). Case–control studies are thereby studies requiring interaction with the study participants.

### Assessment of study quality

All eligible articles underwent a quality assessment of their methodological quality using the Joanna Briggs Institute (JBI) critical appraisal tools for case–control and cohort studies (54). The appraisal checklist has 10 criteria for case–control and 11 for cohort studies. Every question answered with a “yes” received a score of 1, while a “no” scored 0 and “unclear” or “not applicable” also received 0 (**Supplementary Material**, pages 12–15). Prior to the critical appraisal of the articles, we systematically checked the articles for overlaps by study population and by risk factors.

### Statistical analyses

We performed random-effects meta-analyses in order to estimate pooled effect sizes (ES) with their respective 95% CIs. Funnel plots and Egger’s test were employed to assess potential publication bias (55). *I*^2^ statistic was calculated to quantify the heterogeneity of the results between studies. *I*^2^ values of 0% were considered to represent “no heterogeneity”, 1%–35% as “low heterogeneity”, 36%–55% as “moderate”, 56%–70% as “substantial”, and above 71% as “considerable” heterogeneity (56). Analyses were conducted by combining both case–control and cohort studies and separately by study design (case–control vs. cohort studies/nested case–control studies). The combined analysis of cohort and case–control studies was presented as the primary focus, unless otherwise stated. The rationale to combine these studies in the primary analysis was based on the limited number of available studies for several risk factors, with the aim of maximizing statistical power and providing a more comprehensive synthesis of the evidence. In addition, while acknowledging that the different designs have their own strengths and weaknesses, all included studies were conducted with the same objective to identify risk factors, hence combined in the first step and stratified in the second step to explore consistency across study designs. Meta-analyses were only performed when at least two studies were available for a given risk factor, and assessments of publication bias were conducted only when at least three studies were included. For risk factors with only one available study, the results were described narratively. Analyses were conducted using STATA^®^ software, version 15.1 (College Station, TX, USA) using a nominal significance level of 0.05.

## Results

A total of 3,772 unique records were retrieved and screened, leading to the evaluation of 96 full texts. Among these, 75 articles from 59 independent studies (31 articles from 22 case–control studies and 44 articles from 37 cohort studies) met the study inclusion criteria (**Figure 1**; **Tables 1**, **2**).

**Table 2:** Characteristics of the 31 case-control studies included in the systematic review and meta-analysis, sorted by country.

First author	Country	Date of diagnoses	Age (years)	Risk factor	Exposure assessment	Outcome ascertainment	Solid tumor type	Cases	Control	
Latin America										
Rios et al., 2018 (46)	Peru	2012–2015	<18	Parental age	Self-administered questionnaire and hospital records	Hospital records	Retinoblastoma, hepatoblastoma, bone and soft tissue	166	332
Europe										
Hug et al., 2010 (100)	Germany	1992–1994	<15	Parental occupational exposure to extremely low frequency magnetic fields (ELF-MF)	Self-administered questionnaire and telephone interview	Cancer registry	Bone and soft tissue	600	2,382	
Schüz and Forman, 2007 (101)	Germany	1992–1994	<14	Birth weight by gestational age	Self-administered questionnaire and telephone interview	Cancer registry	Bone and soft tissue	541	2,057	
Schüz et al., 2007 (102)	Germany	1992–1994	<14	Medication use during pregnancy	Self-administered questionnaire and telephone interview	Cancer registry	Bone and soft tissue	234	2,057	
Schüz et al., 1999 (103)	Germany	1992–1994	<15	Parental smoking	Self-administered questionnaire and telephone interview	Cancer registry	Bone and soft tissue	217	3,659	
Hardell and Dreifaldt, 2001 (104)	Sweden	1988–1991	<15	Breast feeding	Medical records	Cancer registry	Retinoblastoma, hepatoblastoma, bone and soft tissue	181	860	
Sorahan et al., 1997 (105)	UK	1953–1955	<16	Parental smoking	Face-to-face interview	Birth registry	Bone	293	293	
Sorahan and Lancashire, 2004 (49)	UK	1953–1955, 1971–1976, 1977–1981	<16	Parental smoking	Face-to-face interview	Birth registry	Hepatoblastoma	43	5,777	
Smith et al., 2009 (106)	UK	1991–1996	<15	Birth weight	Face-to-face interview	Cancer registry	Retinoblastoma, hepatoblastoma, and germ cell	3,651	6,337	
Pang et al., 2003 (107)	UK	1991–1994	<15	Parental smoking	Face-to-face interview	Cancer registry	Retinoblastoma, hepatoblastoma, germ cell, bone, and soft tissue tumors	1,378	7,629	
Rajaraman et al., 2011 (108)	UK—England and Wales	1992–1996	<15	Diagnostic radiation	Face-to-face interview	Histopathology review database or individual consultant	Retinoblastoma, hepatoblastoma, and soft tissue	240	4,857	
North America										
Shu et al., 1995 (109)	United States and Canada	1982–1989	<15	Multiple risk factors	Self-administered questionnaire	Children’s cancer group	Germ cell	105	639	
Chen et al., 2005 (110)	USA	1993–2001	<15	Parental smoking and drinking	Self-administered questionnaire and telephone interview	Children’s cancer group	Germ cell	274	421	
Chen et al., 2006 (111)	USA	1993–2001	<15	Exposure to residential pesticides, chemicals, dusts, fumes, and metals	Self-administered questionnaire and telephone interview	Children’s cancer group	Germ cell	272	418	
Musselman et al., 2011 (112)	USA	2000–2008	<6	Birth characteristics	Telephone interview	Children’s Oncology Group	Germ cell	222	336	
Musselman et al., 2013 (113)	USA	2000–2008	<6	Maternal pregnancy events	Telephone interview	Children’s Oncology Group	Hepatoblastoma	383	387	
Janitz et al., 2017 (114)	USA	2000–2008	<6	Parental occupational exposures	Telephone interview	Children’s Oncology Group	Hepatoblastoma	383	387	
Johnson et al., 2008 (115)	USA and Canada	2000–2008	<16	Maternal vitamin intake	Telephone interview	Children’s Oncology Group	Germ cell	278	423	
Johnson et al., 2013 (116)	USA and Canada	2000–2008	<6	Parental tobacco and alcohol consumption	Telephone interview	Children’s Oncology Group	Hepatoblastoma	383	387	
Omidakhsh et al., 2017 (117)	USA and Canada	2006–2012	<16	Residential pesticide exposures	Telephone interview	Children’s Oncology Group	Retinoblastoma	282	564	
Omidakhsh et al., 2018 (118)	USA and Canada	2006–2012	<16	Parental occupational exposures	Telephone interview	Children’s Oncology Group	Retinoblastoma	187	157	
Heck et al., 2015 (119)	USA and Canada	2006–2011	<15	Perinatal characteristics	Telephone interview	Children’s Oncology Group	Retinoblastoma	282	146	
Azary et al., 2016 (120)	USA and Canada	2011–2013	<15	Parental smoking and alcohol consumption	Telephone interview	Children’s Oncology Group	Retinoblastoma	488	424	
Lombardi et al., 2015 (121)	USA and Canada	2006–2012	<16	Maternal diet	Telephone interview	Cancer registry	Retinoblastoma	163	136	
Abdolahi et al., 2013 (122)	USA and Canada	1998–2006	<14	Paternal occupational exposures	Telephone interview	Cancer registry	Retinoblastoma	135	245	
John et al., 1991 (123)	USA—Colorado	1976–1983	<15	Parental smoking	Face-to-face and telephone interview	Cancer registry	Soft tissue	26	196	
Yeazel et al., 1997 (124)	USA—California	1982–1989	<2	Birth weight	Self-administered questionnaire	Children’s cancer group	Soft tissue	531	816	
Savitz and Kaune, 1993 (125)	USA—Colorado	1976–1983	<15	Residential wire code	Face-to-face and telephone interview	Cancer registry	Soft tissue	32	260	
Savitz and Ananth, 1994 (126)	USA—Colorado	1976–1983	<15	Birth characteristics	Face-to-face and telephone interview	Cancer registry	Soft tissue	26	212	
Savitz et al., 1988 (127)	USA—Colorado	1976–1983	<15	Magnetic fields	Face-to-face and telephone interview	Cancer registry	Soft tissue	32	26	
Savitz and Feingold, 1989 (128)	USA—Colorado	1976–1983	<15	Traffic density	Face-to-face and telephone interview	Cancer registry	Soft tissue	32	262	

**Figure 1:**
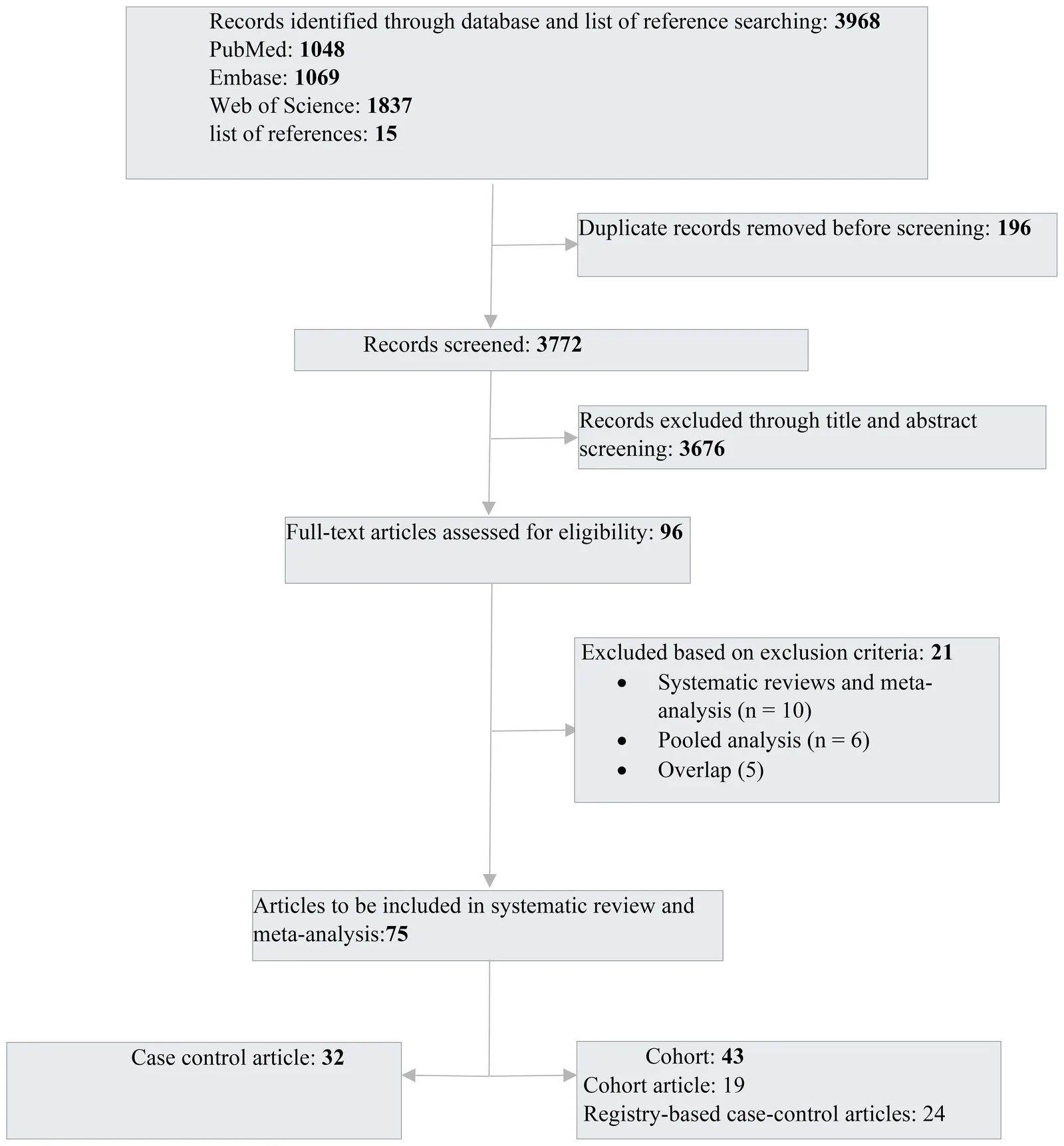
Preferred reporting items for systematic reviews and meta-analyses flow diagram outlining the article selection.

**Table 1:** Characteristics of the 44 cohort studies and registry-based case-control studies included in the systematic review and meta-analysis, sorted by country.

First author	Study design	Country	Date of diagnoses	Age (years)	Risk factor	Data source	Outcome ascertainment	Solid tumor type	Cases	Study population	Follow-up (years)
Oceania											
Stavrou et al., 2009 (57)	CO	Australia	1994–2005	<13	Maternal smoking	Midwives’ data collection	Cancer registry	Retinoblastoma, hepatoblastoma, and germ cell	260	979,809	NA
Asia											
Heck et al., 2020 (58)	CO	China–Taiwan	2004–2014	<12	Gestational risk factors	Hospital records	Cancer registry	Retinoblastoma, hepatoblastoma, and germ cell	614	2,079,037	NA
Huang et al., 2022 (59)	RBCC	China–Taiwan	2004–2014	<12	Gestational diabetes	Hospital records	Cancer registry	Retinoblastoma, hepatoblastoma, and germ cell	2,160	2,076,877	NA
Weng et al., 2022 (60)	CO	China–Taiwan	2004–2017	<14	Assisted reproductive technology (ART)	National databases	Cancer registry	Retinoblastoma and hepatoblastoma	273	2,308,016	11
Cha et al., 2011 (61)	CO	Korea	1995–2006	<12	Birth characteristics	Birth database	Death database	Hepatoblastoma, retinoblastoma, and soft tissue	189	6,479,406	11
Europe											
Momen et al., 2016 (62)	CO	Denmark	1996–2008	<15	Maternal smoking	National patient register	Cancer registry	Retinoblastoma and bone	160	888,556	15
Rios et al., 2024 (63)	CO	France	2010–2021	<15	ART	French National Mother–Child Register	French national registry of childhood cancer	Retinoblastoma and soft tissue	21	8,526,306	10.9
Volk et al., 2020 (64)	RBCC	Denmark	1968–2016	<19	Parental occupational exposure	Employment history from civil registration system	Cancer registry	Hepatoblastoma and retinoblastoma	64	1,116	18
Schüz et al., 2015 (65)	CO	Denmark	1973–2010	<20	Birth order	Population and birth registers	Cancer registry	Retinoblastoma, hepatoblastoma, bone, and soft tissue	1,068	2,461,283	20
Contreras et al., 2017 (66)	RBCC	Denmark	1968–2015	<16	Parental age	Birth certificates	Cancer registry	Retinoblastoma, germ cell, and soft tissue	1,310	585,594	15
Hall et al., 2020 (67)	RBCC	Denmark	1968–2016	<17	Occupational livestock or animal dust	Birth registry and parental occupational history	Cancer registry	Retinoblastoma and soft tissue	32	6,393	16
Hall et al., 2021 (68)	RBCC	Denmark	1968–2015	<16	Parental occupation	Birth registry and parental occupational history	Cancer registry	Germ cell tumor	178	4,355	15
Sundh et al., 2014 (69)	CO	Denmark	1968–2004	<15	ART	Hospital records	Cancer registry	Retinoblastoma, hepatoblastoma, soft tissue, bone, and germ cell	138	450,215	9.5 mean
Momen et al., 2014 (70)	CO	Denmark, Sweden, and Finland	1973–2007	<15	Delivery by caesarean section	Birth registry	Cancer registers	Retinoblastoma, hepatoblastoma, bone, and germ cell	1,515	7,029,843	15
Seppälä et al., 2021 (71)	RBCC	Finland	1996–2014	<20	Preterm birth	Medical records	Cancer registry	Retinoblastoma, hepatoblastoma, soft tissue, bone, and germ cell	140	1,888	19
de Fine Licht et al., 2012 (72)	RBCC	Nordic countries	1985–2006	<15	Multiple risk factors	Birth registry	Cancer registry	Hepatoblastoma	155	775	14
Stephansson et al., 2011 (73)	RBCC	Nordic countries	1967–2006	<15	Multiple risk factors	Birth registry	Cancer registry	Germ cell	152	1,520	14
Bjørge et al., 2013 (74)	RBCC	Nordic countries	1967–2010	<15	Fetal growth	Birth registry	Cancer registries and in direct reports from pediatric, oncology, and neurosurgery hospitals	Retinoblastoma, bone, and soft tissue	2,779	172,422	14
Mortensen et al., 2016 (75)	CO	Norway	1999–2010	<15	Folic acid in pregnancy	Birth registry	Cancer registry	Bone and soft tissue	189	687,406	6
Pershagen et al., 1992 (76)	CO	Sweden	1982–1987	<6	Maternal smoking	Birth registry	Cancer registry	Soft tissue	15	148,917	5
Yip et al., 2006 (77)	CO	Sweden	1961–2000	<15	Parental age	Multi-generation register	Cancer registry	Retinoblastoma	574	4.3 million	14
Rossides et al., 2022 (78)	RBCC	Sweden	1960–2015	<20	Parental occupational exposure to hydrocarbon solvents and engine exhaust fumes	Employment history and job exposure matrix	Cancer registry	Retinoblastoma and hepatoblastoma	1,161^a^	172,194 ^a^	19
Coste et al., 2020 (79)	CO	Switzerland	1990–2015	<16	Parental occupational exposure to pesticides	Census records/CLIC-JEM	Cancer registry	Bone, soft tissue, and germ cell	593	3.5 million	15
MacCarthy et al., 2009 (80)	RBCC	UK	1962–1999	<15	Paternal occupational exposures	Occupational and birth records	National registry	Retinoblastoma	1,318	1,318	14
Williams et al., 2013 (81)	CO	UK	1992–2008	<15	ART	Human Fertilization and Embryology Authority records	Cancer registry	Bone and germ cell	35	106,013	7
Williams et al., 2018 (82)	CO	UK	1992–2008	<15	ART	Human Fertilization and Embryology Authority records	Cancer registry	Retinoblastoma and hepatoblastoma	12	12,137	8
Dockerty et al., 2001 (83)	RBCC	UK	1968–1986	<15	Parental age	Birth records	National Registry of Childhood Tumors	Retinoblastoma	415	10,868	14
Fear et al., 1998 (84)	CO	UK	1959–1990	<15	Occupational exposure to pesticide	Death certificates	National Statistics	Bone and soft tissue	66	167,703	14
Kendall et al., 2020 (85)	RBCC	UK	1962–2010	<15	Paternal occupational exposures	Birth/employment records and job exposure matrix	National registry of Childhood Tumors	Bone and soft tissue	5,369	5,380	14
North America											
Marcoux et al., 2022 (86)	CO	Canada—Quebec	2006–2019	<14	Gestational diabetes	Hospital records	Cancer registry	Retinoblastoma, hepatoblastoma, and soft tissue	61	1,030,537	14
Spector et al., 2019 (87)	CO	USA	2004–2012	<10	*In vitro* fertilization	Hospital records	Cancer registry	Retinoblastoma, hepatoblastoma, soft tissue, and germ cell	59	275,686	8
Hall et al., 2019 (88)	RBCC	USA—California	1984–2013	<6	Prenatal exposure to air toxics	Birth certificates and air measurement	Cancer registry	Germ cell	243	147,100	5
Thompson et al., 2022 (45)	RBCC	USA—California	1988–2013	<16	Prenatal pesticide exposure	Addresses on birth certificate and geocode	Cancer registry	Retinoblastoma	335	123,166	15
Williams et al., 2021a (89)	RBCC	USA—Minnesota	1976–2014	<15	Caesarean section	Birth certificate	Cancer registry	Retinoblastoma, hepatoblastoma, bone, and soft tissue	3,166^a^	20,589	14
Williams et al., 2021b (90)	RBCC	USA—Minnesota, California, New York, Texas, and Washington	1976–2014	<15	Birth characteristics	Birth certificate	Cancer registry	Hepatoblastoma and soft tissue	16,411^a^	69,816	14
McLaughlin et al., 2006 (91)	RBCC	USA—New York	1985–2001	<6	Maternal and infant birth characteristics	Birth certificates	Cancer registry	Hepatoblastoma	58	116	5
Shakeel et al., 2020 (92)	RBCC	USA and Canada	1998–2006	<13	Medical radiation	Dose estimation using X-ray energy	Children’s Oncology Group	Retinoblastoma	482	404	12
Contreras et al., 2016 (93)	RBCC	USA—California	1988–2013	<6	Gestational diabetes	Birth records	Cancer registry	Retinoblastoma and hepatoblastoma	3,508	270,147	5
Morimoto et al., 2016 (94)	RBCC	USA—California	1988–2011	<20	Birth characteristics	Birth certificates	Cancer registry	Soft tissue	722	2,888	19
Heck et al., 2012 (95)	RBCC	USA—California	1988–2007	<6	Perinatal characteristics	Birth certificate	Cancer registry	Retinoblastoma	609	209,051	5
Heck et al., 2013 (96)	RBCC	USA—California	1988–2007	<6	Birth characteristics	Birth certificates	Cancer registry	Hepatoblastoma	261	218,277	5
Kumar et al., 2018 (97)	RBCC	USA—Texas	2003–2009	<5	Maternal residential proximity to major roadways	Texas roadway network StratMap	Cancer registry	Retinoblastoma and hepatoblastoma	144	2,855	4
Flower et al., 2004 (98)	CO	USA—Iowa and North Carolina	1975–1998	<19	Paternal occupational exposures to pesticides	Birth certificate	Cancer registry	Retinoblastoma, bone, soft tissue, and germ cell	50	17,357	18
Carozza et al., 2009 (99)	RBCC	USA—Texas	1990–1998	<15	Pesticides	Digital orthophoto quadrangle (DOQ) data	Cancer registry	Germ cell	696	1,802	14

CO, cohort studies; RBCC, registry-based case–control studies; NA, not available.

^a^Case/study population combined with other risk factors.

Among all of the articles that met the criteria, 47% (*n* = 35) were carried out in North America. Europe came next with 45% (*n* = 34), followed by Asia with 5% (*n* = 4) and Oceania and Latin America with 1% (*n* = 1) each, respectively. There were no eligible studies in Africa. All publications that met the criteria were in English language.

### Study bias and quality assessment

The 75 articles critically appraised for quality using the JBI tools were of good quality (84%). The case–control studies had a slightly higher average score (86%) compared to cohort studies (81%). The least ranked case–control study scored 7 out of 10 points, while for the cohort study it was 7 out of 11 points. Thus, all screened articles appraised were included in the final analysis (**Supplementary Material**, pages 12–15).

### Retinoblastoma

Low birth weight (<2,500 g) showed a borderline association with the risk of retinoblastoma (ES 1.27, 95% CI 0.99–1.63) based on estimates from one case–control study and three cohort studies. The RR for ART was elevated, but with a wide confidence interval, based on estimates from five cohort studies which were consistently above 1.00, except for the study by Sundh and co-authors (69). Gestational age (<37 and >40 weeks) and gestational diabetes also showed elevated risks, but with wide confidence intervals. Breastfeeding (≥6 months), C-section, “small and large for gestational age” (SGA and LGA), high birth weight (>4,000 g), younger parents (<25 years), and older parents (>35 years) were not associated with retinoblastoma risk. The funnel plots were symmetric for the risk factors assessed (**Figure 2**; **Table 3**; **Supplementary Material**, pages 16–21), suggesting no publication bias.

**Figure 2:**
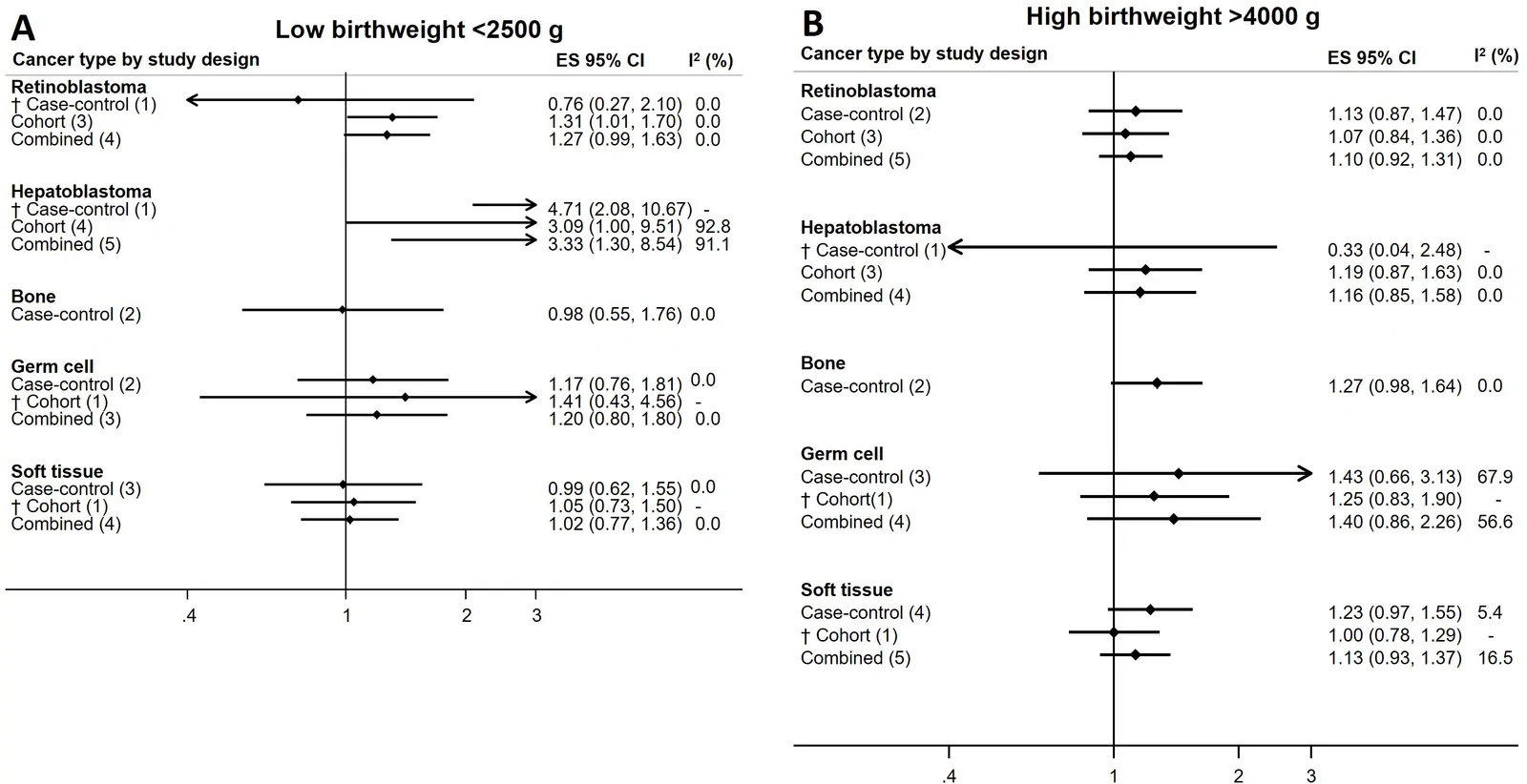
Meta-analysis of pooled effect sizes (ES) of exposure to **(A)** low (<2,500 g) and **(B)** high (>4,000 g) birth weight compared with normal birth weight (2.5–4 kg) for the risk of various childhood cancer types and heterogeneity by study design. †, risk estimate not ES, as only one study was identified.

**Table 3:** Meta-analysis of pooled effect sizes (ES) of birth and parental characteristics, and paternal occupational exposures for the risk of childhood cancer and heterogeneity (*I*^2^) between studies by study design.

Risk factors	Study design	Retinoblastoma			*n*	*I*^2^ (%)	Hepatoblastoma			*n*	*I*^2^ (%)	Bone			*n*	*I*^2^ (%)	Germ cell			*n*	*I*^2^ (%)	Soft tissue			*n*	*I*^2^ (%)
		ES [95% CI]					ES [95% CI]					ES [95% CI]					ES [95% CI]					ES [95% CI]				
ART	Cohort	1.27	0.75	2.16	5	44.5	2.82	1.82	4.39	4	0.0						1.18	0.62	2.23	4	20.0	1.29	0.89	1.87	4	0.0
Caesarean section	Case–control											0.50	0.21	1.19	1	–	1.31	0.82	2.07	1	–	0.85	0.50	1.45	1	–
	Cohort	0.91	0.54	1.55	3	50.5	1.47	1.20	1.79	4	0.0	0.91	0.47	1.78	2	61.9	1.00	0.57	1.73	1	–	0.77	0.44	1.35	1	–
	Combined											0.78	0.42	1.42	3	62.1	1.17	0.82	1.67	2	0.0	0.81	0.55	1.19	2	0.0
Gestational age <37 weeks	Case–control																1.20	0.80	1.90	1	–	6.10	1.60	23.40	1	–
	Cohort	1.43	0.89	2.29	4	49.1	1.90	0.92	0.94	7	84.9						1.11	0.74	1.66	3	0.0	0.87	0.66	1.15	2	0.0
	Combined																1.15	0.85	1.54	4	0.0	1.41	0.60	3.31	3	75.5
Gestational age >40 weeks	Case–control																1.00	0.60	1.50	1	–	0.40	0.10	1.80	1	–
	Cohort	1.39	0.58	3.32	2	43.1	1.13	0.82	1.56	4	0.0						1.10	0.66	1.83	1	–	0.82	0.68	0.98	1	–
	Combined																1.04	0.74	1.47	2	0.0	0.81	0.68	0.97	2	0.0
SGA	Case–control	1.10	0.53	2.10	1	–						0.87	0.31	2.47	2	76.5						1.06	0.72	1.57	2	0.0
	Cohort	1.07	0.83	1.39	1	–	1.76	1.28	2.43	2	0.0											1.00	0.76	1.32	1	–
	Combined	1.07	0.84	1.37	2	0.0																1.02	0.81	1.28	3	0.0
LGA	Case–control	1.09	0.83	1.43	1	–						1.03	0.62	1.72	2	0.0						1.26	0.45	3.50	2	75.3
	Cohort	0.77	0.33	1.80	1	–	1.51	0.86	2.65	2	0.0											0.94	0.72	1.23	1	–
	Combined	1.06	0.81	1.37	2	0.0																1.10	0.66	1.85	3	61.8
Maternal age <25	Case–control						1.02	0.07	14.12	1	–						1.00	0.80	1.40	1	–					
	Cohort	1.15	0.83	1.59	4	0.0	1.40	0.90	2.18	5	47.2						1.02	0.66	0.66	2	0.0	0.84	0.68	1.04	3	
	Combined						1.40	0.93	2.10	6	34.7						1.01	0.80	1.27	3	0.0					0.0
Maternal age >35	Case–control	1.08	0.55	2.10	2	0.0	4.35	0.31	60.38	1	–						1.10	0.70	1.60	1	–	1.59	0.45	8.59	1	–
	Cohort	1.19	0.82	1.74	4	17.4	1.95	1.43	2.66	2	0.0						1.40	0.93	2.11	2	0.0	0.63	0.38	1.04	1	–
	Combined	1.15	0.85	1.57	6	0.0	1.97	1.45	2.68	3	0.0						1.24	0.93	1.66	3	0.0	0.76	0.37	1.60	2	26.3
Paternal age <25	Case–control																1.10	0.70	1.70	1	–					
	Cohort	0.85	0.56	1.29	4	46.7	1.11	0.65	1.91	4	43.1						0.79	0.44	1.44	1	–					
	Combined																0.98	0.69	1.39	2	0.0					
Paternal age >35	Case–control	1.30	0.50	3.60	1	0.0	0.10	0.01	1.80	1	–						1.10	0.90	1.30	1	–	0.39	0.12	1.30	1	–
	Cohort	0.97	0.71	1.33	4	0.0	1.34	1.07	1.67	6	0.0						0.77	0.45	1.30	2	47.0	1.14	0.78	1.66	1	–
	Combined	0.99	0.74	1.34	5	0.0	1.33	0.99	1.79	7	25.5						0.92	0.64	1.32	3	57.8	0.78	0.29	2.13	2	64.7
Gestational diabetes	Case–control																									
	Cohort	1.35	0.93	1.96	2	0.0	1.35	0.86	2.14	3	0.0															
	Combined																									
Paternal occupational exposure																										
Paternal occupational exposure to wood dust	Cohort	1.26	0.74	2.15	2	0.0																				
Paternal occupational exposure to paint	Case–control	1.04	0.35	3.13	2	46.5																				
	Cohort	6.00	0.73	276.00	1	–																				
	Combined	1.33	0.43	4.15	3	43.2																				
Paternal occupational exposure to gasoline fumes	Case–control						1.29	0.64	2.60	2	0.0															
Paternal occupational livestock/animal exposure	Cohort											0.99	0.44	2.22	2	56.2										

The ES for maternal smoking during pregnancy was elevated but with a wide confidence interval, including 1.00 for the risk of retinoblastoma. This was based on the combined estimates from two cohort studies and two case–control studies. Paternal exposure to smoking during preconception/pregnancy and the risk of retinoblastoma did not show evidence of an association based on estimates from two case–control studies only.

Paternal occupational exposure to pesticides during preconception/pregnancy was associated with the risk of retinoblastoma (ES 1.55, 95% CI 1.09–2.21) based on estimates from two case–control and two cohort studies. No heterogeneity was observed across studies. Similarly, maternal residential exposure to pesticides was associated with the risk of retinoblastoma in one cohort study (45). Paternal occupational exposure to wood dust showed an elevated risk of retinoblastoma based on estimates from two cohort studies only. Similarly, the ES of paternal occupational exposure to paint was elevated, but with a wide confidence interval based on estimates from two case–control studies and one cohort study. Maternal medical radiation history of upper and lower gastrointestinal series was associated with retinoblastoma (RR = 1.9, 95% CI: 1.1–3.2 and RR = 6.9, 95% CI: 2.9–16.4, respectively) in one cohort study (92) (**Figures 3**, **4**; **Table 3**; **Supplementary Material**, pages 22 and 23).

**Figure 3:**
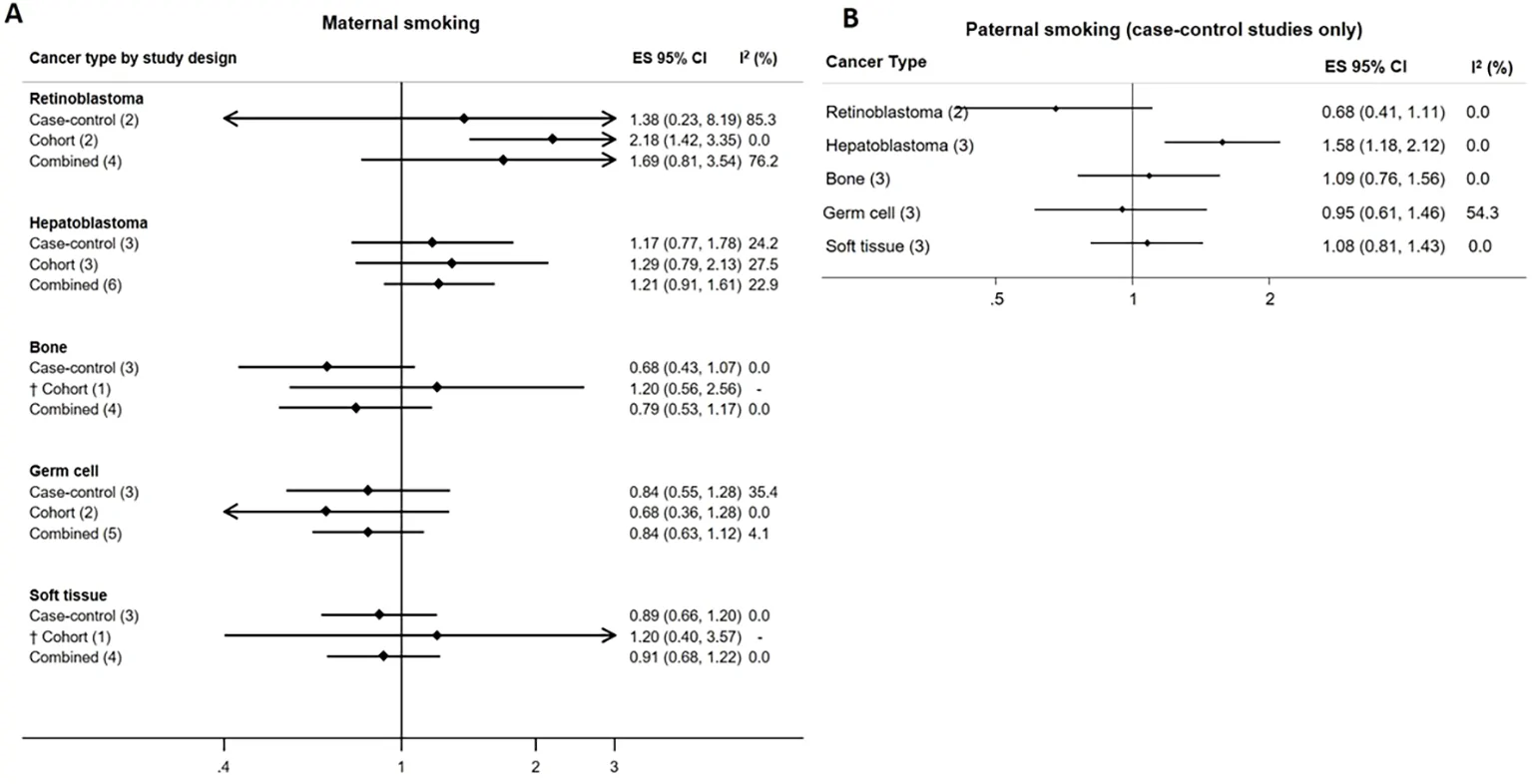
Meta-analysis of pooled effect sizes (ES) of exposure to **(A)** maternal smoking and **(B)** paternal smoking for the risk of various childhood cancer types and heterogeneity by study design. †, risk estimate not ES, as only one study was identified.

**Figure 4:**
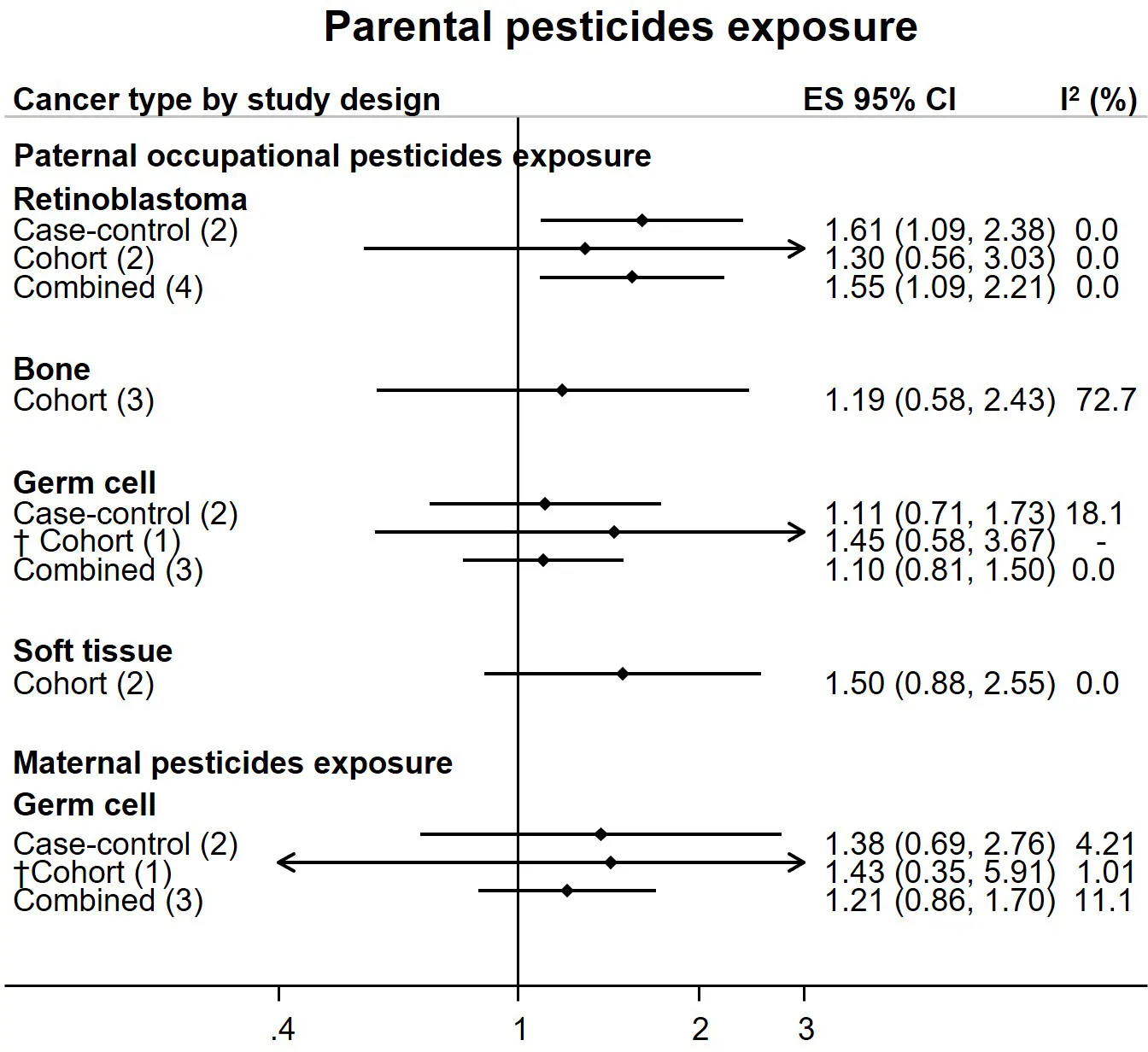
Meta-analysis of pooled effect sizes (ES) of parental exposure to pesticides for the risk of various childhood cancer types and heterogeneity by study design. †, risk estimate not ES, as only one study was identified.

### Hepatoblastoma

Low birth weight (<2,500 g) showed a threefold association with the risk of hepatoblastoma (ES 3.33 CI 1.30–8.54) based on combined estimates from one case–control study and four cohort studies. However, heterogeneity across the studies was considerable (*I*^2^ = 91.1%). ART was twofold associated with the risk of hepatoblastoma (ES 2.82, 95% CI 1.82–4.39) based on estimates from four cohort studies, with a consistent magnitude above 2.00. No heterogeneity across studies was observed. C-section was associated with an increased risk of hepatoblastoma (ES 1.47, 95% CI 1.20–1.79) based on estimates from four cohort studies, showing a consistent magnitude of association. Moderate heterogeneity was observed across studies. SGA was associated with the risk of hepatoblastoma (RR 1.76, 95% CI 1.28–2.43) based on estimates from two cohort studies. Older paternal age (>35 years) showed a borderline association with hepatoblastoma (ES 1.33, 95% CI 0.99–1.79) based on estimates from one case–control and six cohort studies. Similarly, older maternal age (ES 1.97, 95% CI 1.45–2.68) was associated with hepatoblastoma based on one case–control study and two cohort studies. Gestational age (<37 weeks) showed an elevated risk of hepatoblastoma, but with a wide confidence interval, based on estimates from seven cohort studies only. Heterogeneity across studies was considerable. LGA and gestational diabetes also showed elevated risks of hepatoblastoma, but with wide confidence intervals. These were based on two and three cohort studies, respectively. The RR of younger mothers (age <25 years) was elevated for the risk of hepatoblastoma, with a wide confidence interval. Gestational age (>40 weeks), high birth weight (>4,000 g), and young fathers (<25 years) were not associated with the risk of hepatoblastoma based on estimates from four cohort studies, one case–control study and three cohort studies, and four cohort studies, respectively (**Figure 2**; **Table 3**; **Supplementary Material**, pages 24–30).

Paternal smoking during preconception/pregnancy showed an increased risk of hepatoblastoma (RR 1.58, 95% CI 1.18–2.12) based on estimates from three case–control studies, with no heterogeneity across studies. On the contrary, maternal smoking during pregnancy did not show an association with the risk of hepatoblastoma based on estimates from three case–control studies and three cohort studies. Similarly, maternal vitamin intake during pregnancy did not show an association with hepatoblastoma based on estimates from one case–control study (112).

Paternal occupational exposure to gasoline fumes and the risk of hepatoblastoma was ES 1.29, 95% CI 0.64–2.60 based on estimates from two case–control studies. Maternal residential proximity within 500 m of major roadways was not associated with the risk of hepatoblastoma based on estimates from one case–control study conducted in the USA (97). The funnel plots were symmetric for all risk factors assessed except for paternal age (>35 years) (**Figures 3**, **4**; **Table 3**; **Supplementary Material**, pages 24–30).

### Bone tumors

High birth weight (>4,000 g) showed a suggestive association with an increased risk of bone tumors (ES = 1.27, 95% CI 0.98–1.64), although the confidence interval included 1.00, indicating some uncertainty. This was based on estimates from two case–control studies. However, heterogeneity across both studies was substantial (*I*^2^ = 62.1%). ART was associated with an increased risk of bone tumor (RR 2.34, 1.43–3.61) based on estimates from one case–control study. In contrast, no associations were observed between other birth characteristics (C-section, SGA, LGA, and birth weight [<2,500 g]) and the risk of bone tumors.

Parental smoking during the preconception/pregnancy period was not associated with the risk of bone tumors. Similarly, paternal occupational exposure to pesticides or to livestock/animals was not associated with the risk of bone tumors based on estimates from three and two cohort studies, respectively (**Figures 2**–**4**; **Table 3**; **Supplementary Material**, pages 30 and 31).

### Germ cell tumors

Low birth weight (<2,500 g) showed a slightly elevated risk of germ cell tumors, although the wide confidence interval included 1.00, based on estimates from two case–control studies and one cohort study. Similarly, high birth weight (>4,000 g) also showed an elevated risk of germ cell tumors (ES = 1.40), but with a wide confidence interval, based on estimates from three case–control studies and one cohort study. There was no evidence of heterogeneity across studies for low birth weight (<2,500 g), whereas heterogeneity was moderate for high birth weight (>4,000 g). No significant associations were observed for ART, C-section, gestational age (<37 or >40 weeks), and parental age (<25 or >35 years) and the risk of germ cell tumors.

Maternal use of pesticides during pregnancy showed an elevated risk of germ cell tumors, but with a wide confidence interval. This was based on estimates from two case–control studies and one cohort study. A similar elevation was observed for childhood exposure to pesticides and the risk of germ cell tumors (RR 1.60); this was observed in only one cohort study (99). There were no observed associations for parental smoking, maternal alcohol consumption, maternal vitamin intake, and maternal exposure to X-ray during pregnancy and the risk of germ cell tumors, as well as between paternal occupational exposure to pesticides and the risk of germ cell tumors. These were based on estimates from three separate case–control studies, respectively (**Figures 2**-**4**; **Table 3**; **Supplementary Material**, pages 31–34).

### Soft tissue tumors

Short gestational age (<37 weeks) showed an elevated risk of soft tissue tumors (ES 1.41, 95% CI, 0.60–3.31), but with a wide CI, based on one case–control and two cohort studies. No associations were observed between ART (based on four cohort studies), C-section (one case–control and one cohort study), and long gestational age (>40 weeks) (one case–control study and one cohort study) and the risk of soft tissue tumors. Similarly, no associations were observed for SGA and LGA (one case–control and two cohort studies) for birth weight <2,500 g (three case–control studies and one cohort study) and birth weight >4,000 g (four case–control studies and one cohort study). Parental age (<25 or >35 years) was also not associated with the risk of soft tissue tumors based on three cohort studies for maternal age <25 years and one case–control and one cohort study for parents >35 years. Head circumference (<33 or >38 cm) (74) and gestational diabetes (86) were not associated with the risk of soft tissue tumors, each based on one case–control study.

Parental smoking was not associated with the risk of soft tissue tumors based on estimates from three case–control studies and one cohort study. Paternal occupational exposure to pesticides showed an elevated risk of soft tissue tumors (ES 1.50), but with wide confidence interval, based on estimates from two cohort studies. Children exposed to traffic density likewise showed an elevated risk of soft tissue tumor (RR 1.40), albeit based on only one cohort study (128) (**Figures 2**–**4**; **Table 3**; **Supplementary Material**, page 35).

## Discussion

Our systematic review and meta-analysis included 75 articles published between 1988 and 2024 on children diagnosed with retinoblastoma, hepatoblastoma, bone tumors, germ cell tumors, and soft tissue tumors. Among birth characteristics, low birth weight (<2,500 g) was associated with an increased risk of retinoblastoma and hepatoblastoma. C-section, ART, SGA, and older parental age (>35 years) were also associated with the risk of hepatoblastoma. For bone tumors, only high birth weight (>4,000 g) showed a suggestive association. No associations were observed between the risk factors evaluated and the risk of germ cell tumors. Paternal occupational exposure to pesticides was associated with an increased risk of retinoblastoma. Paternal smoking during preconception/pregnancy was associated with an increased risk of hepatoblastoma. Although only a few risk factors were significantly associated with specific childhood cancer types, several others showed elevated ES with wide confidence intervals, indicating non-significant elevations.

The borderline association observed between low birth weight (<2,500 g) and the risk of retinoblastoma may be influenced by other factors, including maternal smoking and alcohol consumption, maternal health conditions, and nutritional status, such as insufficient iron and folic acid supplementation (129–133). However, the pooled estimate for retinoblastoma (ES 1.27, 95% CI 0.99–1.63) was higher than that reported in a recent systematic review and meta-analysis by Rashti et al. in 2025 (134). The authors reported a pooled estimate of ES 0.95 (CI 0.68–1.32) based on one traditional case–control study, one registry-based case–control study, and one pooled analysis which they collectively referred to as “three case–control studies.” In our systematic review and meta-analysis, we included two additional studies (Heck et al., 2012, conducted in California, and Heck et al., 2020, conducted in China—Taiwan) (58, 95) and excluded the pooled analysis from the USA (135), as the California data were updated with 42 cases of retinoblastoma and published separately. Our results showing no association between ART and retinoblastoma align with the systematic review and meta-analysis by Song et al. in 2025 (136).

Some pesticides are genotoxic and can disrupt cell signaling, apoptosis, and cellular proliferation (137). Parental exposure to pesticides may increase the risk of childhood cancer through several windows of susceptibility: preconception, pregnancy, or the postnatal period. Preconception exposures may induce mutations or epigenetic alterations, such as changes in genomic imprinting or DNA methylation, in sperm. Exposures during pregnancy or postnatally may lead to somatic mutations or disrupt hormonal and immunologic function (138–140). In our analysis, paternal occupational exposure to pesticides was associated with an increased risk of retinoblastoma and showed a similarly elevated estimate for soft tissue tumors (ES 1.50), although with a wide confidence interval. Previous systematic reviews and meta-analyses on pesticides and childhood cancer have primarily focused on leukemia and brain tumors, where positive associations have been consistently reported (51, 141–147).

Similar to retinoblastoma, the threefold association observed between low birth weight (<2,500 g) and hepatoblastoma risk may have been influenced by the factors discussed above and other confounders. SGA is strongly correlated with low birth weight (148). In the present systematic review and meta-analysis, its association with hepatoblastoma was of lower magnitude (1.76-fold) compared to low birth weight (<2,500 g). Our findings showing a threefold increased risk of hepatoblastoma among children with low birth weight are consistent with previous epidemiological studies and meta-analyses identifying low birth weight as one of the strongest established risk factors for hepatoblastoma (44, 135). Several biological mechanisms have been proposed to explain this association. Low birth weight and prematurity are frequently associated with neonatal intensive care interventions, including oxygen therapy, with parental nutrition, and with exposure to certain medications. These factors may contribute to oxidative stress, DNA damage, or altered liver development during critical periods of growth (149). None of those proposed mechanisms is widely accepted though.

At least over 8 million children (live births) worldwide are conceived through ART (150), and 2% of children conceived in the USA are through ART (151). Among the childhood cancers reported in this present systematic review and meta-analysis, ART and older parental age (>35) were associated with increased risks of hepatoblastoma. Older parental age may contribute to hepatoblastoma risk through several biologically plausible pathways. Age-related germline mutations, particularly in fathers, increase with each additional year of spermatogonial cell division and may elevate the likelihood of *de novo* variants associated with embryonal tumors (152). Advanced maternal or paternal age has also been linked to epigenetic alterations, including imprinting errors relevant to hepatoblastoma’s association with imprinting disorders such as Beckwith–Wiedemann syndrome (153). Declining oocyte quality and increased chromosomal instability in older mothers may further contribute to developmental susceptibility (154). Additionally, oxidative stress and mitochondrial dysfunction in aging gametes may impair early embryonic liver development (155). While these mechanisms are plausible, they remain speculative, and further mechanistic studies are needed to clarify their relevance.

Children born through C-sections may be at a higher risk of developing malignancies (156). We observed an association between C-section and the risk of hepatoblastoma. Several hypotheses have been proposed to explain the potential link between C-section and childhood cancer. One of the hypotheses suggests that the lack of exposure to the maternal vaginal canal may limit the transfer of the maternal vaginal microbiome to the neonatal gut during a critical window of development, potentially influencing health outcomes later in life (157, 158). Moreover, neonates delivered by C-section do not experience the physiological stress of vaginal delivery, which normally activates the hypothalamic–pituitary–adrenal axis and primes the immune system for future function. This absence of early stress may create a permissive environment for malignancies to develop later in life (89, 159) Previous systematic reviews and meta-analyses have reported associations between C-sections and childhood leukemia (145, 160), Wilms tumor (52, 161), and neuroblastoma (162). Although the direction of association was generally consistent, given the observed heterogeneity across studies, interpreting the findings should be done with appropriate caution. In our study, evidence suggests that paternal smoking during preconception/pregnancy is positively associated with hepatoblastoma, with consistent findings in studies conducted in the UK and US. However, similar associations have previously been reported only in systematic reviews and meta-analyses of other types of childhood cancer (51, 163, 164).

Our findings of an association between high birth weight and an increased risk of bone tumors in children is consistent with previous systematic reviews and meta-analyses (165, 166). The two studies contributing to our pooled estimate were both conducted in Europe (74, 101). However, substantial heterogeneity was observed between studies, which may reflect differences in study populations, methodological approaches, exposure classification, adjustment for confounding factors, and sample sizes. Therefore, the pooled estimate should be interpreted with caution. Pooled estimates for older parental age (>35 years) did not indicate an increased risk of bone tumors, soft tissue tumors, or germ cell tumors. In contrast, Domingues et al. (2022) reported a borderline association between maternal age and childhood germ cell tumors (ES 1.06, 95% CI 1.00–1.12) per 5-year increase. Their estimate is broadly comparable to ours (ES 1.24, 95% CI 0.93–1.66), with differences likely reflecting variations in study inclusion and exclusion criteria (48).

Whether findings rely more on cohort or on case–control studies in the primary meta-analyses has important implications for interpretation. These study designs differ fundamentally in their susceptibility to bias (e.g., recall and selection bias in case–control studies versus loss to follow-up in cohort studies). As a result, combining them may increase statistical heterogeneity. Although the use of a random-effects model partially accounts for between-study variability, residual heterogeneity related to study design remains. Our stratified analyses by study design provide additional context, allowing an assessment of the consistency and robustness of the findings across the two approaches. Where estimates were broadly consistent, this supports the overall validity of the pooled results; however, where differences were observed, findings should be interpreted with caution.

### Strengths and limitations

This systematic review and meta-analysis has several limitations. First, the number of eligible studies was limited, and for many risk factors and cancer types the available evidence was sparse. Most existing systematic reviews and meta-analyses in childhood cancer with adequate study numbers focus primarily on childhood leukemia and brain tumors, leaving other cancer types insufficiently studied. Second, the pooled effect sizes were derived from a combination of case–control and cohort studies, each of which carries inherent sources of bias that cannot be fully ruled out despite methodological precautions. Moreover, some extracted risk estimates were not adjusted for confounding factors, thereby reducing the internal validity of those findings. Finally, the geographical distribution of the included studies was heavily skewed, with most conducted in North America and Europe and only a small number originating from Asia, Oceania, and Latin America. Notably, no eligible studies were identified from Africa, where the exposure levels for several environmental risk factors are comparatively higher. Since the included studies were mainly conducted in high-income countries, the observed associations likely reflect exposure patterns, healthcare contexts, and underlying population characteristics typical of these settings. As a result, generalizing the findings to LMICs’ settings may be inappropriate, given potential differences in exposure prevalence, healthcare access, diagnostic practices, and data availability.

Subgroup and sensitivity analyses by tumor sub-types, publication periods, years of diagnosis, and geographical regions were not feasible because the original studies did not report sufficiently often results according to these specific subgroup classifications (e.g., unilateral vs. bilateral retinoblastoma, gonadal vs. extragonadal germ cell tumors). The overall limited number of available studies substantially restricted the possibility of conducting meaningful stratified analyses by publication periods, years of diagnosis, and geographical regions. These limitations should be considered when interpreting the findings. Our study also has several notable strengths. These include a rigorously designed and well-structured search strategy as well as the separation of case–control and cohort/registry-based studies in the meta-analysis to account for study design differences. Additionally, we stratified the analyses by the timing and source of exposure, distinguishing between paternal, maternal, and childhood exposures, as well as preconception, prenatal, and postnatal exposure windows. This approach enhances the precision and clarity of our findings.

## Conclusion

This review and meta-analysis suggests only a few modifiable risk factors for rare childhood cancers, including paternal smoking for hepatoblastoma and paternal occupational exposure to pesticides for retinoblastoma during preconception/pregnancy. Wide CIs for pooled ES show that the database for evidence synthesis is limited and more and larger studies for rare childhood cancers are needed, with international collaboration to increase sample sizes, which is essential for informing strategies for the primary prevention of childhood cancer.
