# Supplementary material for: Parental and childhood exposures and the risk of retinoblastoma, hepatoblastoma, and germ cell, bone, and soft tissue tumors in children: a systematic review and meta-analysis
Source: Front Oncol. 2026 Sep 16;16:1797553. doi: 10.3389/fonc.2026.1797553 (PMC13623739; doi:10.3389/fonc.2026.1797553)
Supplement: Supplementary file 1 [file SupplementaryFile1.docx]

**Supplemental Online Content**

**Parental and childhood exposures and the risk of retinoblastoma, hepatoblastoma, bone, germ cell and soft tissue tumours in children:**

**a systematic review and meta-analysis**

Felix M. Onyije, Roya Dolatkhah, Ann Olsson, Liacine Bouaoun, and Joachim Schüz

Environment and Lifestyle Epidemiology Branch, International Agency for Research on Cancer (IARC/WHO), 25 avenue Tony Garnier, CS 90627, 69366 LYON CEDEX 07, France

Table 1. Inclusion and exclusion criteria

Table 2. Preferred Reporting Items for Systematic Reviews and Meta-Analyses (PRISMA) checklist

Table 3. Search strategy used in PubMed, Web of Science and Embase databases

Table 4. JBI critical appraisal checklist for cohort studies

Table 5. JBI critical appraisal checklist for case-control studies

Figure 1. Forest and funnel plots of birth and parental characteristics

Figure 2. Forest and funnel plots of parental lifestyle exposures

Figure 3. Forest and funnel plots of chemicals and radiation exposures

**Table 1. Inclusion and exclusion criteria**

| **Criteria** | **Inclusion** | **Exclusion** |
| --- | --- | --- |
| Exposure | - Childhood or parental exposure to environmental pollutants, chemicals, occupational, or lifestyle (e.g. cigarette smoking, alcohol, vitamin supplements etc) - Specific exposure window e.g. preconception, prenatal or postnatal | - Genetic predisposition and other rare risk factors - Assessments without exposure window |
| Study design | - Case-control or cohort studies - Pooled analysis with unique original studies that have not been previously published - Studies with risk estimate and 95% CI   Studies reporting risk estimates for children below < 20 years. | - Ecological or cross-sectional studies - Pooled analysis in which the original studies have been previously published - Studies without risk estimates or 95% CI - Studies reporting risk estimates for children > 20 years. |
| Outcome | - Retinoblastoma, hepatoblastoma, bone, germ cell and soft tissue | - Other types childhood cancers - Non-specified type of cancer (combination of different cancer types or sites) |
| Overlapping studies | - Studies with no overlapping populations by risk factors - Studies with minimal overlapping <40% | - Overlapping study populations by risk factors - Studies with substantial overlapping >40% |
| Search Criteria | No language or publication date restrictions |  |

**Table 2. Preferred Reporting Items for Systematic Reviews and Meta-Analyses (PRISMA) checklist**

| **Section and Topic** | **Item #** | **Checklist item** | **Location where item is reported** |
| --- | --- | --- | --- |
| **TITLE** | | | **Page No.** |
| Title | 1 | Identify the report as a systematic review. | **1** |
| **ABSTRACT** | | |  |
| Abstract | 2 | See the PRISMA 2020 for Abstracts checklist. | **1** |
| **INTRODUCTION** | | |  |
| Rationale | 3 | Describe the rationale for the review in the context of existing knowledge. | **2** |
| Objectives | 4 | Provide an explicit statement of the objective(s) or question(s) the review addresses. | **2** |
| **METHODS** | | |  |
| Eligibility criteria | 5 | Specify the inclusion and exclusion criteria for the review and how studies were grouped for the syntheses. | **3-4, Table 2 and 3** |
| Information sources | 6 | Specify all databases, registers, websites, organisations, reference lists and other sources searched or consulted to identify studies. Specify the date when each source was last searched or consulted. | **3** |
| Search strategy | 7 | Present the full search strategies for all databases, registers and websites, including any filters and limits used. | **Table 1 in supplement 1** |
| Selection process | 8 | Specify the methods used to decide whether a study met the inclusion criteria of the review, including how many reviewers screened each record and each report retrieved, whether they worked independently, and if applicable, details of automation tools used in the process. | **3-4** |
| Data collection process | 9 | Specify the methods used to collect data from reports, including how many reviewers collected data from each report, whether they worked independently, any processes for obtaining or confirming data from study investigators, and if applicable, details of automation tools used in the process. | **4** |
| Data items | 10a | List and define all outcomes for which data were sought. Specify whether all results that were compatible with each outcome domain in each study were sought (e.g. for all measures, time points, analyses), and if not, the methods used to decide which results to collect. | **3-4** |
|  | 10b | List and define all other variables for which data were sought (e.g. participant and intervention characteristics, funding sources). Describe any assumptions made about any missing or unclear information. | **3-4** |
| Study risk of bias assessment | 11 | Specify the methods used to assess risk of bias in the included studies, including details of the tool(s) used, how many reviewers assessed each study and whether they worked independently, and if applicable, details of automation tools used in the process. | **4** |
| Effect measures | 12 | Specify for each outcome the effect measure(s) (e.g. risk ratio, mean difference) used in the synthesis or presentation of results. | **3** |
| Synthesis methods | 13a | Describe the processes used to decide which studies were eligible for each synthesis (e.g. tabulating the study intervention characteristics and comparing against the planned groups for each synthesis (item #5)). | **4** |
|  | 13b | Describe any methods required to prepare the data for presentation or synthesis, such as handling of missing summary statistics, or data conversions. | **3-4** |
|  | 13c | Describe any methods used to tabulate or visually display results of individual studies and syntheses. | **4** |
|  | 13d | Describe any methods used to synthesize results and provide a rationale for the choice(s). If meta-analysis was performed, describe the model(s), method(s) to identify the presence and extent of statistical heterogeneity, and software package(s) used. | **3** |
|  | 13e | Describe any methods used to explore possible causes of heterogeneity among study results (e.g. subgroup analysis, meta-regression). | **3-5** |
|  | 13f | Describe any sensitivity analyses conducted to assess robustness of the synthesized results. | **5** |
| Reporting bias assessment | 14 | Describe any methods used to assess risk of bias due to missing results in a synthesis (arising from reporting biases). | **3 and** **Table 4 in supplement 1** |
| Certainty assessment | 15 | Describe any methods used to assess certainty (or confidence) in the body of evidence for an outcome. | **4-5** |
| **RESULTS** | | |  |
| Study selection | 16a | Describe the results of the search and selection process, from the number of records identified in the search to the number of studies included in the review, ideally using a flow diagram. | **4-5** |
|  | 16b | Cite studies that might appear to meet the inclusion criteria, but which were excluded, and explain why they were excluded. | **4-5** |
| Study characteristics | 17 | Cite each included study and present its characteristics. | **9-15** |
| Risk of bias in studies | 18 | Present assessments of risk of bias for each included study. | **12 and**  **Table 4 in supplement 1** |
| Results of individual studies | 19 | For all outcomes, present, for each study: (a) summary statistics for each group (where appropriate) and (b) an effect estimate and its precision (e.g. confidence/credible interval), ideally using structured tables or plots. | **10-13 and supplement 1** |
| Results of syntheses | 20a | For each synthesis, briefly summarise the characteristics and risk of bias among contributing studies. | **10 and 12** |
|  | 20b | Present results of all statistical syntheses conducted. If meta-analysis was done, present for each the summary estimate and its precision (e.g. confidence/credible interval) and measures of statistical heterogeneity. If comparing groups, describe the direction of the effect. | **10-13 and supplement 1** |
|  | 20c | Present results of all investigations of possible causes of heterogeneity among study results. | **10-13 and supplement 1** |
|  | 20d | Present results of all sensitivity analyses conducted to assess the robustness of the synthesized results. | **14 and**  **Table 5 in Supplement 1** |
| Reporting biases | 21 | Present assessments of risk of bias due to missing results (arising from reporting biases) for each synthesis assessed. | **5-6** |
| Certainty of evidence | 22 | Present assessments of certainty (or confidence) in the body of evidence for each outcome assessed. | **12** |
| **DISCUSSION** | | |  |
| Discussion | 23a | Provide a general interpretation of the results in the context of other evidence. | **15-23** |
|  | 23b | Discuss any limitations of the evidence included in the review. | **24-25** |
|  | 23c | Discuss any limitations of the review processes used. | **24-25** |
|  | 23d | Discuss implications of the results for practice, policy, and future research. | **25-26** |
| **OTHER INFORMATION** | | |  |
| Registration and protocol | 24a | Provide registration information for the review, including register name and registration number, or state that the review was not registered. | **NIL** |
|  | 24b | Indicate where the review protocol can be accessed, or state that a protocol was not prepared. | **3** |
|  | 24c | Describe and explain any amendments to information provided at registration or in the protocol. | **Nil** |
| Support | 25 | Describe sources of financial or non-financial support for the review, and the role of the funders or sponsors in the review. | **25** |
| Competing interests | 26 | Declare any competing interests of review authors. | **25** |
| Availability of data, code and other materials | 27 | Report which of the following are publicly available and where they can be found: template data collection forms; data extracted from included studies; data used for all analyses; analytic code; any other materials used in the review. | **Included in the publication** |

**Table 3. Search strategy used in PubMed, Web of Science and Embase databases**

| **Search No** | **Query** | | **Search Details for PubMed Query** | **Results** |
| --- | --- | --- | --- | --- |
| 1 | "Child*" OR "infan*" OR "New-born" OR "pediatric" OR "paediatric" OR "Childhood" OR "Adolescence" OR "Teenage*" OR "Youth*" | | "child*"[All Fields] OR "infan*"[All Fields] OR "New-born"[All Fields] OR "pediatric"[All Fields] OR "paediatric"[All Fields] OR "Childhood"[All Fields] OR "Adolescence"[All Fields] OR "teenage*"[All Fields] OR "youth*"[All Fields] | **3,824,000** |
| 2 | "Environmental Pollution"[Mesh] OR "Environmental Exposure" OR "Occupational Exposure"[Mesh]OR "Occupational Exposure" OR "Prenatal exposure" OR "Maternal exposure" OR "Paternal exposure" OR "Residential exposure" OR "household exposure" OR "domestic exposure" OR "Indoor exposure" OR "Outdoor exposure" OR "Smok*" OR "Tobacco" OR "Cigarette" OR, "Radiation*" OR "birth weight" OR "Chemical exposure" OR "lifestyle" OR "electromagnetic fields"[MeSH Terms] OR "non-ionising radiation" OR "radiation" OR "phototherapy" OR "Breast feeding" OR "Birth characteristics" OR "Electromagnetic fields" OR "Alcohol" OR "Folic acid" OR "Vitamins" OR "Cesarean Section" OR "Smoking" OR "Paint" OR "Coffee" OR "Tea" OR "Livestock" OR "Birth weight" OR "Parity" OR "Birth order" OR "Gestation" OR "Age" OR "ART" OR "Pesticides" | | (("Environmental Pollution"[MeSH Terms] OR "Environmental Exposure"[All Fields] OR "Occupational Exposure"[MeSH Terms] OR "Occupational Exposure"[All Fields] OR "Prenatal exposure"[All Fields] OR "Maternal exposure"[All Fields] OR "Paternal exposure"[All Fields] OR "Residential exposure"[All Fields] OR "household exposure"[All Fields] OR "domestic exposure"[All Fields] OR "Indoor exposure"[All Fields] OR "Outdoor exposure"[All Fields] OR "smok*"[All Fields] OR "Tobacco"[All Fields] OR "cigarette ("[All Fields])) AND "radiation*"[All Fields]) OR "Birth weight"[All Fields] OR "Chemical exposure"[All Fields] OR "lifestyle"[All Fields] OR "Electromagnetic fields"[MeSH Terms] OR "non-ionising radiation"[All Fields] OR "radiation"[All Fields] OR "phototherapy"[All Fields] OR "Breast feeding"[All Fields] OR "Birth characteristics"[All Fields] OR "Electromagnetic fields"[All Fields] OR "Alcohol"[All Fields] OR "Folic acid"[All Fields] OR "Vitamins"[All Fields] OR "Cesarean Section"[All Fields] OR "Smoking"[All Fields] OR "Paint"[All Fields] OR "Coffee"[All Fields] OR "Tea"[All Fields] OR "Livestock"[All Fields] OR "Birth weight"[All Fields] OR "Parity"[All Fields] OR "Birth order"[All Fields] OR "Gestation"[All Fields] OR "Age"[All Fields] OR "ART"[All Fields] OR "Pesticides"[All Fields] | **8,730,149** |
| 3 | "Case-Control Stud*"[Mesh] OR "Case-Control Stud*" OR "cohort" OR "Epidemiology"[SH] "observational study" | | ("case-control stud*"[MeSH Terms] OR "case-control stud*"[All Fields] OR "cohort"[All Fields] OR "Epidemiology"[MeSH Subheading]) AND "observational study"[All Fields] | **118,206** |
| 4 | "Brain" OR "spinal cord" OR "Neuroblastoma" OR "Wilms tumor" OR "Wilms tumour" OR "Lymphoma" OR "Hodgkin lymphoma" OR "non-Hodgkin lymphoma" OR "Germ cell" OR "Soft tissue" OR "Rhabdomyosarcoma" OR "Retinoblastoma" OR "osteosarcoma" OR "sarcoma" OR "Hepatoblastoma" "Neoplasms"[Mesh] OR "cancer*" OR "cancerous" OR "carcinoma" OR "neoplasm*" OR "tumor*" OR "tumour*" OR "malignan*" | | (("Brain"[All Fields] OR "spinal cord"[All Fields] OR "Neuroblastoma"[All Fields] OR "Wilms tumor"[All Fields] OR "Wilms tumour"[All Fields] OR "Lymphoma"[All Fields] OR "Hodgkin lymphoma"[All Fields] OR "non-hodgkin lymphoma"[All Fields] OR "Germ cell"[All Fields] OR "Soft tissue"[All Fields] OR "Rhabdomyosarcoma"[All Fields] OR "Retinoblastoma"[All Fields] OR "osteosarcoma"[All Fields] OR "sarcoma"[All Fields] OR "Hepatoblastoma"[All Fields]) AND "Neoplasms"[MeSH Terms]) OR "cancer*"[All Fields] OR "cancerous"[All Fields] OR "carcinoma"[All Fields] OR "neoplasm*"[All Fields] OR "tumor*"[All Fields] OR "tumour*"[All Fields] OR "malignan*"[All Fields] | **5,032,012** |
| 5 | #1 AND #2 AND #3 AND #4 | | ("child*"[All Fields] OR "infan*"[All Fields] OR "New-born"[All Fields] OR "pediatric"[All Fields] OR "paediatric"[All Fields] OR "Childhood"[All Fields] OR "Adolescence"[All Fields] OR "teenage*"[All Fields] OR "youth*"[All Fields]) AND ((("Environmental Pollution"[MeSH Terms] OR "Environmental Exposure"[All Fields] OR "Occupational Exposure"[MeSH Terms] OR "Occupational Exposure"[All Fields] OR "Prenatal exposure"[All Fields] OR "Maternal exposure"[All Fields] OR "Paternal exposure"[All Fields] OR "Residential exposure"[All Fields] OR "household exposure"[All Fields] OR "domestic exposure"[All Fields] OR "Indoor exposure"[All Fields] OR "Outdoor exposure"[All Fields] OR "smok*"[All Fields] OR "Tobacco"[All Fields] OR "cigarette ("[All Fields])) AND "radiation*"[All Fields]) OR "Birth weight"[All Fields] OR "Chemical exposure"[All Fields] OR "lifestyle"[All Fields] OR "Electromagnetic fields"[MeSH Terms] OR "non-ionising radiation"[All Fields] OR "radiation"[All Fields] OR "phototherapy"[All Fields] OR "Breast feeding"[All Fields] OR "Birth characteristics"[All Fields] OR "Electromagnetic fields"[All Fields] OR "Alcohol"[All Fields] OR "Folic acid"[All Fields] OR "Vitamins"[All Fields] OR "Cesarean Section"[All Fields] OR "Smoking"[All Fields] OR "Paint"[All Fields] OR "Coffee"[All Fields] OR "Tea"[All Fields] OR "Livestock"[All Fields] OR "Birth weight"[All Fields] OR "Parity"[All Fields] OR "Birth order"[All Fields] OR "Gestation"[All Fields] OR "Age"[All Fields] OR "ART"[All Fields] OR "Pesticides"[All Fields]) AND (("case-control stud*"[MeSH Terms] OR "case-control stud*"[All Fields] OR "cohort"[All Fields] OR "Epidemiology"[MeSH Subheading]) AND "observational study"[All Fields]) AND ((("Brain"[All Fields] OR "spinal cord"[All Fields] OR "Neuroblastoma"[All Fields] OR "Wilms tumor"[All Fields] OR "Wilms tumour"[All Fields] OR "Lymphoma"[All Fields] OR "Hodgkin lymphoma"[All Fields] OR "non-hodgkin lymphoma"[All Fields] OR "Germ cell"[All Fields] OR "Soft tissue"[All Fields] OR "Rhabdomyosarcoma"[All Fields] OR "Retinoblastoma"[All Fields] OR "osteosarcoma"[All Fields] OR "sarcoma"[All Fields] OR "Hepatoblastoma"[All Fields]) AND "Neoplasms"[MeSH Terms]) OR "cancer*"[All Fields] OR "cancerous"[All Fields] OR "carcinoma"[All Fields] OR "neoplasm*"[All Fields] OR "tumor*"[All Fields] OR "tumour*"[All Fields] OR "malignan*"[All Fields]) | **1,469** |
|  |  | **Filters** |  |  |
| 6 | #1 AND #2 AND #3 AND #4 | Abstract | (("child*"[All Fields] OR "infan*"[All Fields] OR "New-born"[All Fields] OR "pediatric"[All Fields] OR "paediatric"[All Fields] OR "Childhood"[All Fields] OR "Adolescence"[All Fields] OR "teenage*"[All Fields] OR "youth*"[All Fields]) AND ((("Environmental Pollution"[MeSH Terms] OR "Environmental Exposure"[All Fields] OR "Occupational Exposure"[MeSH Terms] OR "Occupational Exposure"[All Fields] OR "Prenatal exposure"[All Fields] OR "Maternal exposure"[All Fields] OR "Paternal exposure"[All Fields] OR "Residential exposure"[All Fields] OR "household exposure"[All Fields] OR "domestic exposure"[All Fields] OR "Indoor exposure"[All Fields] OR "Outdoor exposure"[All Fields] OR "smok*"[All Fields] OR "Tobacco"[All Fields] OR "cigarette ("[All Fields])) AND "radiation*"[All Fields]) OR "Birth weight"[All Fields] OR "Chemical exposure"[All Fields] OR "lifestyle"[All Fields] OR "Electromagnetic fields"[MeSH Terms] OR "non-ionising radiation"[All Fields] OR "radiation"[All Fields] OR "phototherapy"[All Fields] OR "Breast feeding"[All Fields] OR "Birth characteristics"[All Fields] OR "Electromagnetic fields"[All Fields] OR "Alcohol"[All Fields] OR "Folic acid"[All Fields] OR "Vitamins"[All Fields] OR "Cesarean Section"[All Fields] OR "Smoking"[All Fields] OR "Paint"[All Fields] OR "Coffee"[All Fields] OR "Tea"[All Fields] OR "Livestock"[All Fields] OR "Birth weight"[All Fields] OR "Parity"[All Fields] OR "Birth order"[All Fields] OR "Gestation"[All Fields] OR "Age"[All Fields] OR "ART"[All Fields] OR "Pesticides"[All Fields]) AND (("case-control stud*"[MeSH Terms] OR "case-control stud*"[All Fields] OR "cohort"[All Fields] OR "Epidemiology"[MeSH Subheading]) AND "observational study"[All Fields]) AND ((("Brain"[All Fields] OR "spinal cord"[All Fields] OR "Neuroblastoma"[All Fields] OR "Wilms tumor"[All Fields] OR "Wilms tumour"[All Fields] OR "Lymphoma"[All Fields] OR "Hodgkin lymphoma"[All Fields] OR "non-hodgkin lymphoma"[All Fields] OR "Germ cell"[All Fields] OR "Soft tissue"[All Fields] OR "Rhabdomyosarcoma"[All Fields] OR "Retinoblastoma"[All Fields] OR "osteosarcoma"[All Fields] OR "sarcoma"[All Fields] OR "Hepatoblastoma"[All Fields]) AND "Neoplasms"[MeSH Terms]) OR "cancer*"[All Fields] OR "cancerous"[All Fields] OR "carcinoma"[All Fields] OR "neoplasm*"[All Fields] OR "tumor*"[All Fields] OR "tumour*"[All Fields] OR "malignan*"[All Fields])) AND (fha[Filter]) | **1,459** |
| 7 | #1 AND #2 AND #3 AND #4 | Abstract, Humans | (("child*"[All Fields] OR "infan*"[All Fields] OR "New-born"[All Fields] OR "pediatric"[All Fields] OR "paediatric"[All Fields] OR "Childhood"[All Fields] OR "Adolescence"[All Fields] OR "teenage*"[All Fields] OR "youth*"[All Fields]) AND ((("Environmental Pollution"[MeSH Terms] OR "Environmental Exposure"[All Fields] OR "Occupational Exposure"[MeSH Terms] OR "Occupational Exposure"[All Fields] OR "Prenatal exposure"[All Fields] OR "Maternal exposure"[All Fields] OR "Paternal exposure"[All Fields] OR "Residential exposure"[All Fields] OR "household exposure"[All Fields] OR "domestic exposure"[All Fields] OR "Indoor exposure"[All Fields] OR "Outdoor exposure"[All Fields] OR "smok*"[All Fields] OR "Tobacco"[All Fields] OR "cigarette ("[All Fields])) AND "radiation*"[All Fields]) OR "Birth weight"[All Fields] OR "Chemical exposure"[All Fields] OR "lifestyle"[All Fields] OR "Electromagnetic fields"[MeSH Terms] OR "non-ionising radiation"[All Fields] OR "radiation"[All Fields] OR "phototherapy"[All Fields] OR "Breast feeding"[All Fields] OR "Birth characteristics"[All Fields] OR "Electromagnetic fields"[All Fields] OR "Alcohol"[All Fields] OR "Folic acid"[All Fields] OR "Vitamins"[All Fields] OR "Cesarean Section"[All Fields] OR "Smoking"[All Fields] OR "Paint"[All Fields] OR "Coffee"[All Fields] OR "Tea"[All Fields] OR "Livestock"[All Fields] OR "Birth weight"[All Fields] OR "Parity"[All Fields] OR "Birth order"[All Fields] OR "Gestation"[All Fields] OR "Age"[All Fields] OR "ART"[All Fields] OR "Pesticides"[All Fields]) AND (("case-control stud*"[MeSH Terms] OR "case-control stud*"[All Fields] OR "cohort"[All Fields] OR "Epidemiology"[MeSH Subheading]) AND "observational study"[All Fields]) AND ((("Brain"[All Fields] OR "spinal cord"[All Fields] OR "Neuroblastoma"[All Fields] OR "Wilms tumor"[All Fields] OR "Wilms tumour"[All Fields] OR "Lymphoma"[All Fields] OR "Hodgkin lymphoma"[All Fields] OR "non-hodgkin lymphoma"[All Fields] OR "Germ cell"[All Fields] OR "Soft tissue"[All Fields] OR "Rhabdomyosarcoma"[All Fields] OR "Retinoblastoma"[All Fields] OR "osteosarcoma"[All Fields] OR "sarcoma"[All Fields] OR "Hepatoblastoma"[All Fields]) AND "Neoplasms"[MeSH Terms]) OR "cancer*"[All Fields] OR "cancerous"[All Fields] OR "carcinoma"[All Fields] OR "neoplasm*"[All Fields] OR "tumor*"[All Fields] OR "tumour*"[All Fields] OR "malignan*"[All Fields])) AND ((fha[Filter]) AND (humans[Filter])) | **1,416** |
| 8 | #1 AND #2 AND #3 AND #4 | Abstract, Humans,  Child: birth-18 years | (("child*"[All Fields] OR "infan*"[All Fields] OR "New-born"[All Fields] OR "pediatric"[All Fields] OR "paediatric"[All Fields] OR "Childhood"[All Fields] OR "Adolescence"[All Fields] OR "teenage*"[All Fields] OR "youth*"[All Fields]) AND ((("Environmental Pollution"[MeSH Terms] OR "Environmental Exposure"[All Fields] OR "Occupational Exposure"[MeSH Terms] OR "Occupational Exposure"[All Fields] OR "Prenatal exposure"[All Fields] OR "Maternal exposure"[All Fields] OR "Paternal exposure"[All Fields] OR "Residential exposure"[All Fields] OR "household exposure"[All Fields] OR "domestic exposure"[All Fields] OR "Indoor exposure"[All Fields] OR "Outdoor exposure"[All Fields] OR "smok*"[All Fields] OR "Tobacco"[All Fields] OR "cigarette ("[All Fields])) AND "radiation*"[All Fields]) OR "Birth weight"[All Fields] OR "Chemical exposure"[All Fields] OR "lifestyle"[All Fields] OR "Electromagnetic fields"[MeSH Terms] OR "non-ionising radiation"[All Fields] OR "radiation"[All Fields] OR "phototherapy"[All Fields] OR "Breast feeding"[All Fields] OR "Birth characteristics"[All Fields] OR "Electromagnetic fields"[All Fields] OR "Alcohol"[All Fields] OR "Folic acid"[All Fields] OR "Vitamins"[All Fields] OR "Cesarean Section"[All Fields] OR "Smoking"[All Fields] OR "Paint"[All Fields] OR "Coffee"[All Fields] OR "Tea"[All Fields] OR "Livestock"[All Fields] OR "Birth weight"[All Fields] OR "Parity"[All Fields] OR "Birth order"[All Fields] OR "Gestation"[All Fields] OR "Age"[All Fields] OR "ART"[All Fields] OR "Pesticides"[All Fields]) AND (("case-control stud*"[MeSH Terms] OR "case-control stud*"[All Fields] OR "cohort"[All Fields] OR "Epidemiology"[MeSH Subheading]) AND "observational study"[All Fields]) AND ((("Brain"[All Fields] OR "spinal cord"[All Fields] OR "Neuroblastoma"[All Fields] OR "Wilms tumor"[All Fields] OR "Wilms tumour"[All Fields] OR "Lymphoma"[All Fields] OR "Hodgkin lymphoma"[All Fields] OR "non-hodgkin lymphoma"[All Fields] OR "Germ cell"[All Fields] OR "Soft tissue"[All Fields] OR "Rhabdomyosarcoma"[All Fields] OR "Retinoblastoma"[All Fields] OR "osteosarcoma"[All Fields] OR "sarcoma"[All Fields] OR "Hepatoblastoma"[All Fields]) AND "Neoplasms"[MeSH Terms]) OR "cancer*"[All Fields] OR "cancerous"[All Fields] OR "carcinoma"[All Fields] OR "neoplasm*"[All Fields] OR "tumor*"[All Fields] OR "tumour*"[All Fields] OR "malignan*"[All Fields])) AND ((fha[Filter]) AND (humans[Filter]) AND (allchild[Filter])) | **1,048** |
|  |  |  |  |  |
|  | | |  |  |
| **Search No** | | | **Embase Query** | **Results** |
| #1 | | | ('child*' OR 'infan*' OR 'new-born' OR 'pediatric'/mj OR 'paediatric'/mj OR 'childhood'/mj OR 'adolescence'/mj OR 'teenage*' OR 'youth*':kw) AND [article]/lim AND ([child]/lim OR [adolescent]/lim) AND [humans]/lim AND [abstracts]/lim AND [embase]/lim | **993,352** |
| #2 | | | ('environmental exposure'/mj OR 'occupational exposure'/mj OR 'prenatal exposure'/mj OR 'maternal exposure'/mj OR 'paternal exposure'/mj OR 'residential exposure'/mj OR 'household exposure' OR 'domestic exposure' OR 'indoor exposure' OR 'outdoor exposure' OR 'tobacco'/mj OR 'cigarette'/mj OR 'chemical' OR 'lifestyle'/mj OR 'non ionising radiation' OR 'radiation'/mj OR 'phototherapy'/mj OR 'breast feeding'/mj OR 'birth characteristics' OR 'electromagnetic fields'/mj OR 'alcohol'/mj OR 'folic acid'/mj OR 'vitamins'/mj OR 'cesarean section'/mj OR 'smoking'/mj OR 'paint'/mj OR 'coffee'/mj OR 'tea'/mj OR 'livestock'/mj OR 'birth weight'/mj OR 'parity'/mj OR 'birth order'/mj OR 'gestation'/mj OR 'age'/mj OR 'art'/mj OR 'pesticides':kw) AND [article]/lim AND ([child]/lim OR [adolescent]/lim) AND [humans]/lim AND [abstracts]/lim AND [embase]/lim | **39,293** |
| #3 | | | ('case-control stud*' OR 'cohort' OR  'epidemiology'/mj OR 'observational study':kw) AND [article]/lim AND ([child]/lim OR [adolescent]/lim) AND [humans]/lim AND [abstracts]/lim AND [embase]/lim |  |
| #4 | | | (('brain'/mj OR 'spinal cord'/mj OR 'neuroblastoma'/mj OR 'wilms tumor'/mj OR 'wilms tumour'/mj OR 'lymphoma'/mj OR 'hodgkin lymphoma'/mj OR 'non-hodgkin lymphoma'/mj OR 'germ cell'/mj OR 'rhabdomyosarcoma'/mj OR 'retinoblastoma'/mj OR 'osteosarcoma'/mj OR 'sarcoma'/mj OR 'hepatoblastoma'/mj) AND 'neoplasms'/mj OR 'cancer*' OR 'cancerous' OR 'carcinoma'/mj OR 'neoplasm*' OR 'tumor*' OR 'tumour*' OR 'malignan*':kw) AND [article]/lim AND ([child]/lim OR [adolescent]/lim) AND [humans]/lim AND [abstracts]/lim AND [embase]/lim | **187,915** |
| #5 | | | #1 AND #2 AND #3 AND #4 | **1,069** |
|  | | |  |  |
| **Search No** | | | **Web of Science Query** | **Results** |
| 1 | | | **ALL=(“Child*” OR “infan*” OR “New-born” OR “pediatric” OR “paediatric” OR “Childhood” OR “Adolescence” OR “Teenage*” OR “Youth*”)** | **4,195,704** |
| 2 | | | **TI=(“Environmental Pollution" OR "Environmental Exposure" OR "Occupational Exposure" OR "Occupational Exposure" OR “Prenatal exposure” OR “Maternal exposure”** OR "Paternal exposure"[All Fields] **OR “Residential exposure” OR “household exposure” OR “domestic exposure” OR “Indoor exposure” OR “Outdoor exposure” OR “Smok*” OR “Tobacco” OR “Cigarette” OR, “Radiation*” OR “birth weight” OR “Chemical exposure” OR “lifestyle” OR "electromagnetic fields" OR "non-ionising radiation" OR "radiation” OR “phototherapy” OR “Breast feeding” OR “Birth characteristics” OR “Electromagnetic fields” OR “Alcohol” OR “Folic acid” OR “Vitamins” OR “Cesarean Section” OR “Smoking” OR “Paint” OR “Coffee” OR “Tea” OR “Livestock” OR “Birth weight” OR “Parity” OR “Birth order” OR “Gestation” OR “Age” OR “ART” OR “Pesticides”)** | **1,574,892** |
| 3 | | | **ALL= ("Case-Control Stud*" OR "Case-Control Stud*” OR “cohort” OR "Epidemiology" OR "observational study")** | **1,917,679** |
| 4 | | | **TI=(“Brain” OR “spinal cord” OR “Neuroblastoma” OR “Wilms tumor” OR “Wilms tumour” OR “Lymphoma” OR “Hodgkin lymphoma” OR “non-Hodgkin lymphoma” OR “Rhabdomyosarcoma” OR “Retinoblastoma” OR “osteosarcoma” OR “sarcoma” OR “Hepatoblastoma” "Neoplasms" OR “cancer*” OR “cancerous” OR “carcinoma” OR “neoplasm*” OR “tumor*” OR “tumour*” OR “malignan*” )** | **3,389,200** |
| 5 | | | **#1 AND #2 AND #3 AND #4** | **1,837** |

**Table 4. JBI critical appraisal checklist for cohort studies**

|  | **First author, year** | Free of selection bias? | Clear exposure measurement? | Valid & reliable exposure measurement? | Identified confounders? | Confounder management strategies stated? | Free of outcome sample? | Valid & reliable outcome measure? | Sufficient follow-up time? | Follow-up complete? | Incomplete follow-up strategies? | Appropriate statistical analysis? | Overall |
| --- | --- | --- | --- | --- | --- | --- | --- | --- | --- | --- | --- | --- | --- |
| 1. | Stavrou et al., 2009 | Y | Y | Y | Y | Y | U | Y | U | N | N | Y | 7 |
| 2. | Heck et al., 2020 | Y | Y | Y | Y | Y | U | Y | N | N | U | Y | 7 |
| 3. | Weng et al., 2022 | Y | Y | Y | Y | Y | U | Y | Y | Y | U | Y | 9 |
| 4. | Cha et al., 2011 | Y | Y | Y | Y | Y | U | Y | Y | Y | U | Y | 9 |
| 5. | Momen et al., 2016 | Y | Y | Y | Y | Y | U | Y | Y | Y | U | Y | 9 |
| 6. | Rios et al., 2024 | Y | Y | Y | Y | Y | U | Y | Y | Y | U | Y | 9 |
| 7. | Schüz et al., 2015 | Y | Y | Y | Y | Y | U | Y | Y | Y | U | Y | 9 |
| 8. | Sundh et al., 2014 | Y | Y | Y | Y | Y | U | Y | Y | Y | U | Y | 9 |
| 9. | Momen et al., 2014 | Y | Y | Y | Y | Y | U | Y | Y | Y | U | Y | 9 |
| 10. | Mortensen et al., 2016 | Y | Y | Y | Y | Y | U | Y | Y | Y | U | Y | 9 |
| 11. | Pershagen et al., 1992 | Y | Y | Y | Y | Y | U | Y | Y | U | U | N | 7 |
| 12. | Yip et al., 2006 | Y | Y | Y | Y | Y | U | Y | U | U | U | Y | 7 |
| 13. | Coste et al., 2020 | Y | Y | Y | Y | Y | U | Y | Y | Y | U | Y | 9 |
| 14. | Williams et al., 2013 | Y | Y | Y | N | N | U | Y | Y | Y | U | Y | 7 |
| 15. | Williams et al., 2018 | Y | Y | Y | N | N | U | Y | Y | Y | U | Y | 7 |
| 16. | Fear et al., 1998 | Y | Y | Y | Y | Y | U | U | N | N | N | Y | 6 |
| 17. | Marcoux et al., 2022 | Y | Y | Y | Y | Y | U | Y | Y | Y | U | Y | 9 |
| 18. | Spector et al., 2019 | Y | Y | Y | Y | Y | U | Y | Y | Y | U | Y | 9 |
| 19. | Flower et al., 2004 | Y | Y | Y | Y | Y | U | Y | Y | Y | U | Y | 9 |
| a. |  |  |  |  |  |  | **156/209*100** | | | | | | **156 (74.6%)** |
| b. | **Registry-based case-control studies classified as cohort studies** | | | | | | | | | | | | |
|  | **First author, year [reference]** | Free of selection bias? | Appropriate sampling? | The same criteria used for cases and control? | Standard, valid and reliable exposure measurement? | Exposure measured the same way for cases and control? | Identified confounders? | Confounder management strategies stated? | | Valid & reliable outcome measure? | Sufficient exposure time? | Appropriate statistical analysis? | Overall |
| 20. | Huang et al., 2022 | Y | Y | Y | Y | Y | Y | Y | | Y | U | Y | 9 |
| 21. | Volk et al., 2020 | Y | Y | Y | Y | Y | Y | N | | Y | U | Y | 8 |
| 22. | Contreras et al., 2017 | Y | Y | Y | Y | Y | Y | Y | | Y | U | Y | 9 |
| 23. | Hall et al., 2020 | Y | Y | Y | Y | Y | Y | Y | | Y | U | Y | 7 |
| 24. | Hall et al., 2021 | Y | Y | Y | Y | Y | Y | Y | | Y | U | Y | 7 |
| 25. | Seppälä et al., 2021 | Y | Y | Y | Y | Y | Y | Y | | Y | U | Y | 9 |
| 26. | de Fine Licht et al., 2012 | Y | Y | Y | Y | Y | Y | Y | | Y | U | Y | 9 |
| 27. | Stephansson et al., 2011 | Y | Y | Y | Y | Y | Y | Y | | Y | U | Y | 9 |
| 28. | Bjørge et al., 2013 | Y | Y | Y | Y | Y | Y | Y | | Y | U | Y | 9 |
| 29. | Rossides et al., 2022 | Y | Y | Y | Y | Y | Y | Y | | Y | U | Y | 9 |
| 30. | MacCarthy et al., 2009 | Y | Y | Y | Y | Y | Y | Y | | Y | U | Y | 9 |
| 31. | Dockerty et al., 2001 | Y | Y | Y | Y | Y | Y | Y | | Y | U | Y | 9 |
| 32. | Kendall et al., 2020 | Y | Y | Y | Y | Y | Y | Y | | Y | U | Y | 9 |
| 33. | Hall et al., 2019 | Y | Y | Y | Y | Y | Y | Y | | Y | U | Y | 9 |
| 34. | Thompson et al., 2022 | Y | Y | Y | Y | Y | Y | Y | | Y | U | Y | 9 |
| 35. | Williams et al., 2021a | Y | Y | Y | Y | Y | Y | Y | | Y | U | Y | 9 |
| 36. | Williams et al., 2021a | Y | Y | Y | Y | Y | Y | Y | | Y | U | Y | 9 |
| 37. | McLaughlin et al., 2006 | Y | Y | Y | Y | Y | Y | Y | | Y | U | Y | 9 |
| 38. | Shakeel et al., 2020 | Y | Y | Y | Y | Y | Y | Y | | Y | U | Y | 9 |
| 39. | Contreras et al., 2016 | Y | Y | Y | Y | Y | Y | Y | | Y | U | Y | 9 |
| 40. | Morimoto et al., 2016 | Y | Y | Y | Y | Y | Y | Y | | Y | U | Y | 9 |
| 41. | Heck et al., 2012 | Y | Y | Y | Y | Y | Y | Y | | Y | U | Y | 9 |
| 42. | Heck et al., 2013 | Y | Y | Y | Y | Y | Y | Y | | Y | U | Y | 9 |
| 43. | Kumar et al., 2018 | Y | Y | Y | Y | Y | Y | Y | | Y | U | Y | 9 |
| 44. | Carozza et al., 2009 | Y | Y | Y | U | Y | Y | Y | | Y | U | Y | 8 |
|  |  |  |  |  |  |  |  | **219/250*100** | | | | | **219(87.6)** |
|  | **Total percentage** |  |  |  |  |  |  | **74.6+87.9÷ 2** | | | | | **81.1%** |

*Yes=1, No=0, Unclear =0*

**Table 5. JBI critical appraisal checklist for case-control studies**

|  | **First author, year [reference]** | Free of selection bias? | Appropriate sampling? | The same criteria used for cases and control? | Standard, valid and reliable exposure measurement? | Exposure measured the same way for cases and control? | Identified confounders? | Confounder management strategies stated? | Valid & reliable outcome measure? | Sufficient exposure time? | Appropriate statistical analysis? | Overall |
| --- | --- | --- | --- | --- | --- | --- | --- | --- | --- | --- | --- | --- |
| 1. | Rios et al., 2018 | Y | Y | Y | Y | Y | Y | Y | Y | U | Y | 9 |
| 2. | Hug et al., 2010 | Y | Y | Y | Y | Y | Y | Y | Y | U | Y | 9 |
| 3. | Schüz and Forman, 2007 | Y | Y | Y | Y | Y | Y | Y | Y | U | Y | 9 |
| 4. | Schüz et al., 2007 | Y | Y | Y | Y | Y | Y | Y | Y | U | Y | 9 |
| 5. | Schüz et al., 1999 | Y | Y | Y | Y | Y | Y | Y | Y | U | Y | 9 |
| 6. | Hardell and Dreifaldt, 2001 | Y | Y | Y | Y | Y | Y | Y | Y | U | Y | 9 |
| 7. | Sorahan et al., 1997 | Y | Y | Y | Y | Y | Y | Y | Y | U | Y | 9 |
| 8. | Sorahan and Lancashire, 2004 | Y | Y | Y | Y | Y | Y | Y | Y | U | Y | 9 |
| 9. | Smith et al., 2009 | Y | Y | Y | Y | Y | Y | Y | Y | U | Y | 9 |
| 10. | Pang et al., 2003 | Y | Y | Y | Y | Y | Y | Y | Y | U | Y | 9 |
| 11. | Rajaraman et al., 2011 | Y | Y | Y | Y | Y | Y | Y | Y | U | Y | 9 |
| 12. | Shu et al., 1995 | N | N | Y | Y | Y | Y | Y | Y | U | Y | 7 |
| 13. | Chen et al., 2005 | N | N | Y | Y | Y | Y | Y | Y | U | Y | 9 |
| 14. | Chen et al., 2006 | Y | Y | Y | Y | Y | Y | Y | Y | U | Y | 9 |
| 15. | Musselman et al., 2011 | Y | Y | Y | Y | Y | Y | Y | Y | U | Y | 9 |
| 16. | Musselman et al., 2013 | Y | Y | Y | Y | Y | Y | N | Y | U | Y | 8 |
| 17. | Janitz et al., 2017 | Y | Y | Y | Y | Y | Y | Y | Y | U | Y | 9 |
| 18. | Johnson et al., 2008 | Y | Y | Y | Y | Y | Y | Y | Y | U | Y | 9 |
| 19. | Johnson et al., 2013 | Y | Y | Y | Y | Y | Y | Y | Y | U | Y | 9 |
| 20. | Omidakhsh et al., 2017 | Y | Y | Y | Y | Y | Y | Y | Y | U | Y | 9 |
| 21. | Omidakhsh et al., 2018 | Y | Y | Y | Y | Y | Y | Y | Y | U | Y | 9 |
| 22. | Heck et al., 2015 | N | Y | Y | Y | Y | Y | Y | Y | U | Y | 8 |
| 23. | Azary et al., 2016 | Y | Y | Y | Y | Y | Y | Y | Y | U | Y | 9 |
| 24. | Lombardi et al., 2015 | N | Y | Y | Y | Y | Y | Y | Y | U | Y | 8 |
| 25. | Abdolahi et al., 2013 | Y | Y | Y | Y | Y | Y | Y | Y | U | Y | 9 |
| 26. | John et al., 1991 | Y | Y | Y | Y | Y | Y | Y | Y | U | U | 8 |
| 27. | Yeazel et al., 1997 | Y | U | U | Y | Y | Y | Y | Y | U | Y | 7 |
| 28. | Savitz and Kaune, 1993 | N | N | N | Y | Y | Y | Y | Y | U | Y | 7 |
| 29. | Savitz and Ananth, 1994 | N | N | Y | Y | Y | Y | Y | Y | U | Y | 9 |
| 30. | Savitz et al., 1988 | N | N | N | Y | Y | Y | Y | Y | U | Y | 7 |
| 31. | Savitz and Feingold, 1989 | Y | Y | Y | Y | Y | Y | Y | Y | U | Y | 9 |
| a. | **Total (%)** |  |  |  |  |  |  | **267/310*100** | | | | **275(86.1)** |

*Yes=1, No=0, Unclear =0*

**Figure 1.1a Forest plot of birth characteristics and the risk of retinoblastoma**

**Figure 1.1b Forest plot of birth characteristics and the risk of retinoblastoma**

**Figure 1.1c Forest plot of birth characteristics and the risk of retinoblastoma**

**Figure 1.1d Forest plot of birth characteristics and the risk of retinoblastoma**

**Figure 1.2 Funnel plot of ART and the risk of retinoblastoma**

**Figure 1.3 Funnel plot of high birthweight (>4000 g) and the risk of retinoblastoma**

**Figure 1.4a Forest plot of parental characteristics and the risk of retinoblastoma**

**Figure 1.4b Forest plot of parental characteristics and the risk of retinoblastoma**

**Figure 1.4c Forest plot of parental characteristics and the risk of retinoblastoma**

**Figure 1.5 Funnel plot of maternal age and the risk of retinoblastoma**

**Figure 1.6 Funnel plot of paternal age and the risk of retinoblastoma**

**Figure 1. 7a Forest plot of parental smoking, occupational exposure to pesticides, paint and wood duct and the risk of retinoblastoma**

**Figure 1. 7b Forest plot of paternal occupational exposure to pesticides and the risk of retinoblastoma**

**Figure 1. 7b Forest plot of paternal occupational exposure to paint and the risk of retinoblastoma**

**Figure 2.1a Forest plot of birth characteristics and the risk of hepatoblastoma**

**Figure 2.1b Forest plot of birth characteristics and the risk of hepatoblastoma**

**Figure 2.2 Funnel plot of low birthweight (<2500 g) age and the risk of hepatoblastoma**

**Figure 2.3a Forest plot of maternal age, diabetes and smoking and the risk of hepatoblastoma**

**Figure 2.3b Forest plot of maternal age < 25 years and the risk of hepatoblastoma**

**Figure 2.3c Forest plot of maternal age > 35years and the risk of hepatoblastoma**

**Figure 2.3d Forest plot of maternal smoking and the risk of hepatoblastoma**

**Figure 2.4 Funnel plot of maternal smoking and the risk of hepatoblastoma**

**Figure 2.5 Forest plot of paternal smoking and the risk of hepatoblastoma**

**Figure 2.6 Funnel plot of gestational age < 37 weeks and the risk of hepatoblastoma**

**Figure 2.7 Funnel plot of maternal age < 25 years and the risk of hepatoblastoma**

**Figure 2.8 Funnel plot of paternal age >35 years and the risk of hepatoblastoma**

**Figure 3.1 Forest plot of birth characteristic and the risk of bone tumour**

**Figure 3.2 Forest plot of parental exposure to smoking, pesticides and livestock/animal and the risk of bone tumour**

**Figure 4.1 Forest plot of birth characteristics and the risk of germ cell tumour**

**Figure 4.2 Forest plot of parental characteristics and the risk of germ cell tumour**

**Figure 4.3 Forest plot of maternal pesticides and the risk of germ cell tumour**

**Figure 4.4 Forest plot of paternal pesticides and the risk of germ cell tumour**

**Figure 4.5 Forest plot of parental smoking and the risk of germ cell tumour**

**Figure 4.5 Funnel plot of maternal smoking and the risk of germ cell tumour**

**Figure 5.1 Forest plot of birth characteristics and the risk of soft tissue tumour**

**Figure 5.2 Forest plot of birth order and weight and the risk of soft tissue tumour**

**Figure 5.3 Forest plot of maternal smoking and the risk of soft tissue tumour**

**Figure 5.4 Forest plot of parental characteristics and other factors and the risk of soft tissue tumour**

**Figure 5.5 Funnel plot of high birthweight (>4000 g) and the risk of soft tissue tumour**
